# Supplementary material for: Measures and Penalties for Animal Welfare Violations at German Abattoirs: A Compilation of Current Recommendations and Practices
Source: Animals (Basel). 2023 Sep 14;13(18):2916. doi: 10.3390/ani13182916 (PMC10525597; doi:10.3390/ani13182916)

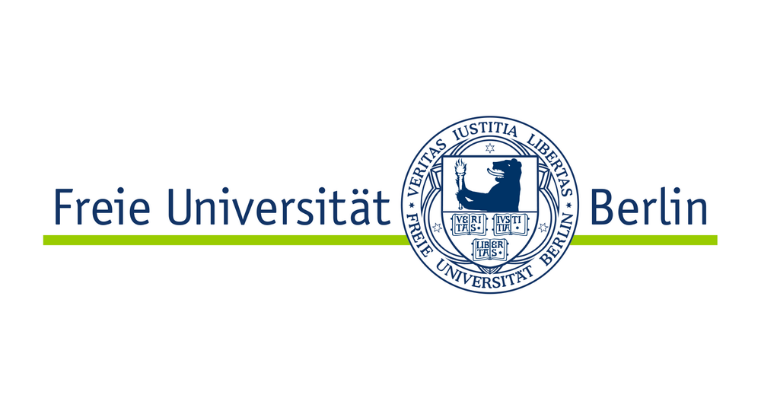


**Measures and Penalties Recommended by Official Veterinarians for Animal Welfare Violations at German Abattoirs**

**Completed October 1st, 2022**


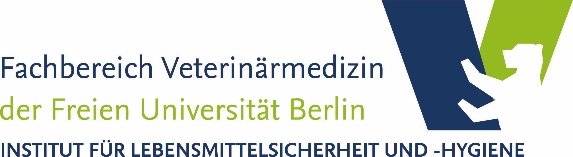


Table of Contents:

[Part 1: Overview of Possible Measures and Penalties for Animal Welfare Violations at Abattoirs 4](#_Toc142823124)

[Part 2: Measures and Penalties Against Violence and Use of Prohibited Driving Aids 7](#_Toc142823125)

[Part 3: Measures and Penalties for Inadequate Housing and Husbandry in Lairage 21](#_Toc142823126)

[Part 4: Measures and Penalties for Violations Related to Inappropriate Handling of Animals with Special Care Needs 33](#_Toc142823127)

[Part 5: Measures and Penalties for Improper Restraint, Stunning, and Bleeding of Animals at Abattoirs 37](#_Toc142823128)

[Part 6: Measures and Penalties for employees performing tasks without an appropriate certificate of competence 51](#_Toc142823129)

Introduction

This document presents an overview of measures and penalties deemed appropriate by official veterinarians (OV) for animal welfare violations at German abattoirs. Additionally, it summarises relevant procedural outcomes within the framework of German and EU legislation. The working group Meat Hygiene of the Institute of Food Safety and Food Hygiene at Freie Universität Berlin developed this list of measures and penalties as part of a research project funded by the German Veterinary Association for Animal Welfare (Tierärztliche Vereinigung für Tierschutz e. V.). This research project involved a collaboration with OV, lawyers, and other professionals responsible for enforcing animal welfare laws and regulations at abattoirs. This document demonstrates which measures and penalties OV consider to be appropriate for the first and repeated violations of regulations outlined in the Regulation (EC) No 1099/2009, the German Animal Welfare Act (Tierschutzgesetz) and the German Ordinance on the Protection of Animals in Connection with Slaughter or Killing (Tierschutzschlacht-Verordnung). Additionally, brief summaries of procedural outcomes are provided for violations during preslaughter handling, stunning, and exsanguination. These findings will be reviewed by legal professionals in the future, in order to contribute to the standardisation of measures following violations nationwide.

Notes on the use of this document

This document is divided into six parts. Part 1 describes general measures that can and/or must be taken in the event of an animal welfare violation. Parts 2-6 deal with animal welfare violations after a slaughter animal’s arrival at the abattoir (preslaughter handling, stunning, and exsanguination).

It should be noted that the violations described in this document are not exhaustive, and other animal welfare violations may occur at abattoirs. This document may be revised in the future, if changes in relevant laws and/or regulations require it, or if the suggestions for improvement proposed by experienced colleagues are based on legal norms.

We hope that this document will assist in the complex task of enforcing animal welfare laws and regulations at abattoirs. If you have any feedback or suggestions for improvement, please feel free to contact us.

Contact Information

Univ.-Prof. Dr. med. vet. Diana Meemken, Dr. med. vet. Susann Langforth, Mag. med. vet. Stephanie Schneidewind

School of Veterinary Medicine

Institute of Food Safety and Food Hygiene

Working Group Meat Hygiene

Königsweg 67, Gebäude 21/22

14163 Berlin

Telephone: +49 30 838 63847

Fax: +49 30 838 4 63847

E-Mail: Diana.Meemken@fu-berlin.de

List of abbreviations

| **Abbreviation** | **Meaning** |
| --- | --- |
| EC | European Commission |
| e.g. | For example |
| vs. | Versus |

List of laws, regulations and legal terms translated from German into English language

| **English** | **German** |
| --- | --- |
| Administrative Procedure Act | Verwaltungsverfahrensgesetz |
| Act on Regulatory Offences | Ordnungswidrigkeitengesetz |
| German Animal Welfare Act | Tierschutzgesetz |
| German Ordinance on the Protection of Animals in Connection with Slaughter or Killing | Tierschutz-Schlachtverordnung |
| Regulatory fine | Bußgeld |
| Regulatory Offence | Ordnungswidrigkeit |

# Part 1: Overview of Possible Measures and Penalties for Animal Welfare Violations at Abattoirs

Measures that should always be taken according to OV:

- Intervention
  - If possible: correct the incorrect method of working/technique or improve the animals’ situation (e.g. separation of incompatible animals when housed together or protection of an animal from avoidable pain, suffering or harm).
- Conduct an oral briefing with the responsible person(s) or request the animal welfare officers and/or the supervisors of the responsible person(s) to conduct an oral briefing.
- Inform the supervisors of the responsible employees and/or animal welfare officers about the incident.
- Investigate the causes for the animal welfare violation, including a discussion with all persons involved, and take corrective actions to rectify the underlying issue.
- Issue an animal protection order that mandates the remedy of deficiencies for the current case and the prevention of danger in the future (see "administrative procedures" in the next section).
- Request a statement from the animal welfare officers describing how a repetition of this offense will be avoided in the future.
- Check to what extent superiors (e.g. animal welfare officers) have fulfilled their obligations to perform checks by requesting documentation and assess the measures taken against past animal welfare violations.
- Carry out more frequent inspections in the affected area(s) of the abattoir.

Further measures that can/must be taken depending on the outcome of the assessment regarding the individual case

1. Administrative procedures

Administrative procedures serve to remedy defects and prevent future dangers. These procedures can take place alongside regulatory or criminal procedures. The following bullet points outline possible administrative procedures, along with relevant pieces of advice:

- Provide a written notice to the responsible person(s), communicating a decision, request, or warning to the recipient.
- Provide a written notice to the operator(s) of the abattoir and the animal welfare officer(s).
- An administrative hearing is held in accordance with Section 28 of the Administrative Procedure Act (Verwaltungsverfahrensgesetz) to prepare an animal protection order.
- An animal protection order can be issued on the basis of Section 16a of the German Animal Welfare Act.
- Specific measures relating to the individual case can be ordered to rectify the underlying issue. If necessary, a deadline can be set, and a sum for periodic penalty payment can be decided upon. For example:
  - If the underlying problem is caused by logistical, personnel, and/or structural deficiencies, request the operator of the abattoir to submit a concept for remedying the issue in the future.
  - If the abattoir's operational self-monitoring is deficient, issue an order to improve operational self-monitoring.
  - If the person responsible for an animal welfare violation intentionally or negligently causes significant pain or suffering to an animal, prohibit this person from working in that position.
  - If employees with a certificate of competence according to Article 21 of Regulation (EC) No 1099/2009 show a lack of competence, knowledge, and/or sense of responsibility to carry out the tasks for which the certificate was issued, temporarily or permanently withdraw the certificate of competence.
  - If staff expertise is deficient, issue an animal protection order for regular training regarding animal welfare standard operating procedures.
  - Review and (if necessary) adapt standard operating procedures.
- Impose a periodic penalty payment if an enforceable order is not complied with. Frameworks for periodic penalty payments can be found in the tables as a reference.

1. Regulatory offence procedures

- Regulatory offences are violations of the law that are not considered serious enough by the legislator to be punished by criminal fines or imprisonment but can still be fined by an administrative authority (source: German Federal Ministry of Justice). Regulatory offence procedures can occur concurrently with administrative and criminal procedures. The following penalties are possible in cases of regulatory offences:
  - Verbal warning of the responsible person(s) according to Section 56 of the Act on Regulatory Offences (Ordnungswidrigkeitengesetz).
  - Cautionary procedure according to Section 56 of the Act on Regulatory Offences with a cautionary fine ranging from €5.00 to €55.00.
  - Proceeding according to Section 130 of the Act on Regulatory Offences against the person(s) responsible for the operation if there are indications that the required supervision to prevent breaches of obligations at the abattoir is intentionally or negligently omitted.
  - Regulatory offence proceeding according to Section 18 of the German Animal Welfare Act with a regulatory fine appropriate for the individual case (considering the amount of pain, suffering, and damage inflicted on the animal, as well as the income of the person responsible). The framework of fines provided in the following tables is a rough guideline. According to Section 18(4) of the German Animal Welfare Act, a regulatory offence can be punished with a fine of up to €25,000.00 in cases of Sections (1), Numbers 1 and 3(a), Numbers 4 to 8, 11, 12, 17, 20, 20a, 22, and 25, (2) as well as (3), Number 1(a) and Number 2(a), and with a fine of up to €5,000.00 in other cases.
  - If a regulatory offence proceeding is being conducted but there are also indications of a criminal offence, there is a duty to hand this matter over to the public prosecutor's office according to Section 41(1) of the Act on Regulatory Offences.
  - The person(s) responsible for the abattoir should be informed about the procedures and penalties in case of (repeated) offences in the future.

1. Criminal procedures

The penal provisions of the Animal Welfare Act can be found in Section 17. Inflicting significant pain or suffering on an animal or prolonged or repetitive significant pain or suffering on a vertebrate out of cruelty is a criminal offence according to Section 17 (a and b, respectively). A criminal complaint must always be filed if there is evidence of an offence according to Section 17 of the German Animal Welfare Act. If the case is not handed over to the public prosecutor's office, criminal liability for obstruction of justice by omission may be considered (Sections 258, 258a, 13 of the German Criminal Code). When criminal charges are filed, the operator(s) of the abattoir should be informed. Administrative offence procedures and criminal charges can take place at the same time of animal protection orders.

Section 40 of the German Criminal Code describes how fines are imposed: A fine is imposed in daily rates. The minimum fine is five and, unless otherwise provided by law, the maximum is 360 full daily rates. The court determines the amount of the daily rate having regard to the offender’s personal and financial circumstances. In doing so, it typically bases its assessment on the average net income which the offender earns or could earn in one day. A daily rate is set at no less than 1 euro and no more than 30,000 euros. The offender’s income and assets and other relevant assessment factors may be estimated when setting the amount of the daily rate. The number and amount of the daily rates are indicated in the decision.

# Part 2: Measures and Penalties Against Violence and Use of Prohibited Driving Aids

**Offense (1):** Use of instruments which administer electric shocks in contravention of Section 5 of the German Ordinance on the Protection of Animals in Connection with Slaughter or Killing, in conjunction with Annex III(1.9) of Regulation (EC) No 1099/2009, where multiple violations occur simultaneously (e.g. repeated, inadequately spaced out administrations of electric shocks in body regions other than the muscles of the hindquarters)

| **Citation of legal/regulatory requirements to protect animals at the time of slaughter/killing** | **Citation of relevant regulatory and criminal penalties** | **Special measures/penalties recommended by OV for the first offence** | **Special measures/penalties recommended by OV if the offence is repeated** | **Judicial decisions/ notes** |
| --- | --- | --- | --- | --- |
| Section 5(1)(1) of the German Ordinance on the Protection of Animals in Connection with Slaughter or Killing in conjunction with Annex III(1.9) of Regulation (EC) No 1099/2009 | According to Section 18(1)(3)(a) of the German Animal Welfare Act in conjunction with Section 16(1)(1) of the German Ordinance on the Protection of Animals in Connection with Slaughter or Killing, any person who deliberately or negligently uses an electric instrument which administers electric shocks in contravention of section 5(1)(1) shall be guilty of a regulatory offence  According to Section 17(2)(a) and/or (b) of the German Animal Welfare Act, any person who commits an offence out of cruelty or if the animal was subjected to prolonged or repeated significant pain or suffering, shall be liable to up to three years imprisonment or a fine | Instruct/train all employees on the proper use of instruments that administer electric shocks in accordance with Section 5 of the German Ordinance on the Protection of Animals in Slaughter or Killing, and Regulation (EC) No 1099/2009. If applicable, the animal welfare officer(s) should be responsible for providing this training  If necessary, a clear sign prohibiting the use of devices that administer electric shocks should be prominently displayed at the abattoir (e.g. pictogram)  Appropriate range for a regulatory fine: €350.00 to €850.00  Investigate whether the operator of the abattoir violated Section 130 of the German Act on Regulatory Offences  File a criminal complaint | If an employee has intentionally or negligently caused significant pain or suffering to an animal, an animal protection order should be issued to prohibit the employee from continuing to work in this or any other relevant position, until they have undergone re-training and have provided proof of acquiring the necessary expertise  Appropriate range for a regulatory fine:  €700.00 to €1,700.00  File a criminal complaint | Punished as criminal offences in past cases:  1) Despite the fact that the cow's head was caught in the fixing device of the killing box, an electric cattle prod was repeatedly used on its back and muzzle. The cow uttered clear vocalizations of pain.  Fine: 40 daily rates.  Source: Stendal District Court,  Penalty order from June 22^nd^, 2020,  Reference number: Cs 444 Js 17063/18  2) Numerous pigs were subjected to electric shocks on their backs, lateral chest, and shoulders, with some receiving up to three shocks. The standard operating procedures clearly stated that only non-electric prods, prodding paddles, and prodding boards could be used as driving aids. Moreover, the stunning gondola was overloaded with up to four fattening pigs, exceeding the limit of two specified in the regulations.  Fine: 90 daily rates.  Source: Kulmbach Local Court,  penalty order from December 27^th^, 2021,  Reference number: unknown |

| **Citation of legal/regulatory requirements to protect animals at the time of slaughter/killing** | **Citation of relevant regulatory and criminal penalties** | **Special measures/penalties recommended by OV for the first offence** | **Special measures/penalties recommended by OV if the offence is repeated** | **Judicial decisions/ notes** |
| --- | --- | --- | --- | --- |
|  |  |  |  | 3) Multiple electric shocks were administered to body parts other than the muscles of the hindquarters (such as the hip, back, and anus), and in some instances, two electric prods were used simultaneously. Additionally, some shocks exceeded a duration of one second.  Fine: 40 daily rates.  Source: Tauberbischofsheim Local Court,  Penalty order from April 25th, 2019,  Reference number: Cs 21 Js 8867/18  4) Multiple applications of an electric cattle prod on body regions other than the hindquarters muscles (such as the back and head) were performed at inappropriate time intervals.  Fine: 30 daily rates.  Source: Tauberbischofsheim Local Court,  Penalty order from April 25th, 2019,  Reference number: Cs 21 Js 8806/18  General note: Instruments which administer electric shocks should be stored in such a way that it is inconvenient to get them. This will increase the use of other aids. |

**Offense (2):** Use of instruments which administer electric shocks in contravention of Section 5 of the German Ordinance on the Protection of Animals in Connection with Slaughter or Killing in conjunction with Annex III(1.9 of Regulation (EC) No 1099/2009, whereby one violation occurs (e.g. use of an electric prod on an animal that is too young, but apart from this, the use complies with animal welfare regulations

| **Citation of legal/regulatory requirements to protect animals at the time of slaughter/killing** | **Citation of relevant regulatory and criminal penalties** | **Special measures/penalties recommended by OV for the first offence** | **Special measures/penalties recommended by OV if the offence is repeated** | **Judicial decisions/ notes** |
| --- | --- | --- | --- | --- |
| Section 5(1)(1) of the German Ordinance on the Protection of Animals in Connection with Slaughter or Killing in conjunction with Annex III(1.9) of Regulation (EC) No 1099/2009 | According to Section 18(1)(3)(a) of the German Animal Welfare Act in conjunction with Section 16(1)(1) of the German Ordinance on the Protection of Animals in Connection with Slaughter or Killing, any person who deliberately or negligently uses an electric instrument which administers electric shocks in contravention of section 5(1)(1) shall be guilty of a regulatory offence  According to Section 17(2)(a) and/or (b) of the German Animal Welfare Act, any person who commits an offence out of cruelty or if the animal was subjected to prolonged or repeated significant pain or suffering, shall be liable to up to three years imprisonment or a fine | Instruct/train all employees on the proper use of instruments that administer electric shocks in accordance with Section 5 of the German Ordinance on the Protection of Animals in Slaughter or Killing, and Regulation (EC) No 1099/2009. If applicable, the animal welfare officer(s) should be responsible for providing this training  If necessary, a clear sign prohibiting the use of devices that administer electric shocks should be prominently displayed at the abattoir (e.g. pictogram)  Appropriate range for a regulatory fine:  €175.00 to €425.00  Investigate whether the operator of the abattoir violated Section 130 of the German Act on Regulatory Offences | If an employee has intentionally or negligently caused significant pain or suffering to an animal, an animal protection order should be issued to prohibit the employee from continuing to work in this or any other relevant position, until they have undergone re-training and have provided proof of acquiring the necessary expertise  Appropriate range for a regulatory fine:  €350.00 to €850.00  File a criminal complaint | General note: Instruments which administer electric shocks should be stored in such a way that it is inconvenient to get it. This will increase the use of other aids. |

**Offense (3):** Striking an animal with a driving stick, particularly against a sensitive body part such as the eye, causing harm or distress

| **Citation of legal/regulatory requirements to protect animals at the time of slaughter/killing** | **Citation of relevant regulatory and criminal penalties** | **Special measures/penalties recommended by OV for the first offence** | **Special measures/penalties recommended by OV if the offence is repeated** | **Judicial decisions/ notes** |
| --- | --- | --- | --- | --- |
| Article 15(1) in conjunction with Annex III(1.8)(a) of Regulation (EC) No 1099/2009 | According to Section 18(3)(2)(a) of the German Animal Welfare Act in conjunction with Section 16(3)(7) of the German Ordinance on the Protection of Animals in Connection with Slaughter or Killing, any person who deliberately or negligently does not ensure that an animal is not beaten or kicked in contravention of Article 15(1) in conjunction with Annex III(1.8)(a) shall be guilty of a regulatory offence  According to Section 17(2)(a) and/or (b) of the German Animal Welfare Act, any person who commits an offence out of cruelty or if the animal was subjected to prolonged or repeated significant pain or suffering, shall be liable to up to three years imprisonment or a fine | Appropriate range for a regulatory fine:  €200.00 to €500.00  Investigate whether the operator of the abattoir violated Section 130 of the German Act on Regulatory Offences  File a criminal complaint | Appropriate range for a regulatory fine:  €400.00 to €1,000.00  If an employee has intentionally or negligently caused significant pain or suffering to an animal, an animal protection order should be issued to prohibit the employee from continuing to work in this or any other relevant position, until they have undergone re-training and have provided proof of acquiring the necessary expertise  If the person concerned no longer demonstrates sufficient competence, knowledge, or sense of responsibility to carry out relevant tasks, their certificate of competence according to Article 21 of Regulation (EC) No 1099/2009 may be temporarily or permanently withdrawn  File a criminal complaint | Punished as criminal offences in past cases:  1) While a bull was being herded through the single file chute, the defendant abruptly struck the animal's right eye with a driving stick, causing significant swelling within minutes.  Fine: €2,100  Source: Oldenburg Higher Regional Court,  Court order from June 14^th^, 2019,  Reference number: 1 Ss 93/19.  2) The defendant repeatedly struck a cow's stomach, rump, and limbs with a driving stick in order to compel the animal to move forward. Moreover, the defendant intentionally hit a limb that was already injured.  A fine of 60 daily rates was imposed for this offence.  Source: Olpe Local Court,  Court verdict from November 11^th^, 2020,  Reference number: 52 Ds 222/20  3) A cow (when trying to get over a barrier in order to regroup with other cows) was subjected to several forceful blows on its back with a stick, intensifying its fear and distress  The offence, together with other offences, was punished with a fine.  Source: Stendal District Court,  Penalty order,  Reference number: Cs 444 Js 15746/18 |

**Offense** **(4):** Kicking an animal

| **Citation of legal/regulatory requirements to protect animals at the time of slaughter/killing** | **Citation of relevant regulatory and criminal penalties** | **Special measures/penalties recommended by OV for the first offence** | **Special measures/penalties recommended by OV for the event of failure to remedy the defect by the deadline or in the event of repeated offence** | **Judicial decisions/ notes** |
| --- | --- | --- | --- | --- |
| Article 15(1) in conjunction with Annex III(1.8)(a) of Regulation (EC) No 1099/2009 | According to Section 18(3)(2)(a) of the German Animal Welfare Act in conjunction with Section 16(3)(7) of the German Ordinance on the Protection of Animals in Connection with Slaughter or Killing, any person who deliberately or negligently does not ensure that an animal is not beaten or kicked in contravention of Article 15(1) in conjunction with Annex III(1.8)(a) shall be guilty of a regulatory offence  According to Section 17(2)(a) and/or (b) of the German Animal Welfare Act, any person who commits an offence out of cruelty or if the animal was subjected to prolonged or repeated significant pain or suffering, shall be liable to up to three years imprisonment or a fine | Appropriate range for a regulatory fine:  €325.00 to €775.00  If the animal is kicking due to structural or technical deficiencies, such as being stuck between two metal posts, an animal protection order should be issued to require the abattoir to make necessary structural changes to correct the underlying issue  Investigate whether the operator of the abattoir violated Section 130 of the German Act on Regulatory Offences  File a criminal complaint | If an employee has intentionally or negligently caused significant pain or suffering to an animal, an animal protection order should be issued to prohibit the employee from continuing to work in this or any other relevant position, until they have undergone re-training and have provided proof of acquiring the necessary expertise  If the person concerned no longer demonstrates sufficient competence, knowledge, or sense of responsibility to carry out relevant tasks, their certificate of competence according to Article 21 of Regulation (EC) No 1099/2009 may be temporarily or permanently withdrawn  Appropriate range for a regulatory fine/periodic penalty payment:  €650.00 to €1,550.00  File a criminal complaint |  |

**Offense (5):** Intentionally dropping a door or a gate onto an animal, causing harm or distress

| **Citation of legal/regulatory requirements to protect animals at the time of slaughter/killing** | **Citation of relevant regulatory and criminal penalties** | **Special measures/penalties recommended by OV for the first offence** | **Special measures/penalties recommended by OV if the offence is repeated** | **Judicial decisions/ notes** |
| --- | --- | --- | --- | --- |
| Article 15(1) in conjunction with Annex III(1.8)(c) of Regulation (EC) No 1099/2009 | According to Section 18(3)(2)(a) of the German Animal Welfare Act in conjunction with Section 16(3)(9) German Ordinance on the Protection of Animals in Connection with Slaughter or Killing, any person who deliberately or negligently fails to ensure that animals are handled in such a way which avoids unnecessary pain or suffering in contravention of Article 15(1) in conjunction with Annex III(1.8)(c) shall be guilty of a regulatory offence  According to Section 17(2)(a) and/or (b) of the German Animal Welfare Act, any person who commits an offence out of cruelty or if the animal was subjected to prolonged or repeated significant pain or suffering, shall be liable to up to three years imprisonment or a fine | Issue an animal protection order which prohibits lowering doors and gates onto animals  Appropriate range for a regulatory fine:  €175.00 to €550.00  Investigate whether the operator of the abattoir violated Section 130 of the German Act on Regulatory Offences | If an employee has intentionally or negligently caused significant pain or suffering to an animal, an animal protection order should be issued to prohibit the employee from continuing to work in this or any other relevant position, until they have undergone re-training and have provided proof of acquiring the necessary expertise  If the person concerned no longer demonstrates sufficient competence, knowledge, or sense of responsibility to carry out relevant tasks, their certificate of competence according to Article 21 of Regulation (EC) No 1099/2009 may be temporarily or permanently withdrawn  Appropriate range for a regulatory fine/periodic penalty payment:  €350.00 to €1,100.00  File a criminal complaint | The following case was treated as a criminal offense and punished accordingly:  A cow was driven from the holding pen into the "killing box" despite another cow already being inside. The driven cow slipped. The accused lowered the slider of the "killing box", which hit the animal's back hard and trapped it. After the accused had raised the slide, the cow was unable to stand up. The accused used an electric prod to shock the cow several times, mostly to its head and neck, although it was clearly not possible for the cow to walk forward. The accused then lowered the slider of the "killing box" again and clamped the animal again. A fine of 60 daily rates was imposed for this offence.  Source: Stendal District Court,  Penalty order from June 22^nd^, 2020,  Reference number: Cs 444 Js 17063/18 |

**Offense (6):** Use of a driving tool with a pointed or sharp-edged end to handle or move an animal, causing potential harm or distress

| **Citation of legal/regulatory requirements to protect animals at the time of slaughter/killing** | **Citation of relevant regulatory and criminal penalties** | **Special measures/penalties recommended by OV for the first offence** | **Special measures/penalties recommended by OV if the offence is repeated** | **Judicial decisions/ notes** |
| --- | --- | --- | --- | --- |
| Article 15(1) in conjunction with Annex III(1.8)(d) of Regulation (EC) No 1099/2009 | According to Section 18(3)(2)(a) of the German Animal Welfare Act in conjunction with Section 16(3)(10) German Ordinance on the Protection of Animals in Connection with Slaughter or Killing, any person who deliberately or negligently does not ensure that a driving aid or other equipment referred to therein is not used in contravention of Article 15(1) in conjunction with Annex III(1.8)(d) shall be guilty of a regulatory offence  According to Section 17(2)(a) and/or (b) of the German Animal Welfare Act, any person who commits an offence out of cruelty or if the animal was subjected to prolonged or repeated significant pain or suffering, shall be liable to up to three years imprisonment or a fine | Appropriate range for a regulatory fine:  €200.00 to €500.00  Investigate whether the operator of the abattoir violated Section 130 of the German Act on Regulatory Offences | Appropriate range for a regulatory fine:  €400.00 to €1,000.00  If an employee has intentionally or negligently caused significant pain or suffering to an animal, an animal protection order should be issued to prohibit the employee from continuing to work in this or any other relevant position, until they have undergone re-training and have provided proof of acquiring the necessary expertise  If the person concerned no longer demonstrates sufficient competence, knowledge, or sense of responsibility to carry out relevant tasks, their certificate of competence according to Article 21 of Regulation (EC) No 1099/2009 may be temporarily or permanently withdrawn  File a criminal complaint | The following violation resulted in the imposition of a regulatory fine:  The act of repeatedly striking a bovine animal's anal area with a pointed stick (fine: €250.00)  Source: Tauberbischofsheim Local Court,  Penalty order from April 25^th^, 2019,  Reference number: Cs 21 Js 8867/18 |

**Offense (7):** Squeezing, rotating or breaking an animal's tail

| **Citation of legal/regulatory requirements to protect animals at the time of slaughter/killing** | **Citation of relevant regulatory and criminal penalties** | **Special measures/penalties recommended by OV for the first offence** | **Special measures/penalties recommended by OV if the offence is repeated** | **Judicial decisions/ notes** |
| --- | --- | --- | --- | --- |
| Article 15(1) in conjunction with Annex III (1.8.)(e) of Regulation (EC) No 1099/2009 | According to Section 18(3)(2)(a) of the German Animal Welfare Act in conjunction with Section 16(3)(11) of the German Ordinance on the Protection of Animals in Connection with Slaughter or Killing, any person who deliberately or negligently does not ensure that the tail of an animal is not squeezed, rotated or broken, or that an animal is not poked in the eyes in contravention of Article 15(1) in conjunction with Annex III(1.8)(e) shall be guilty of a regulatory offence  According to Section 17(2)(a) and/or (b) of the German Animal Welfare Act, any person who commits an offence out of cruelty or if the animal was subjected to prolonged or repeated significant pain or suffering, shall be liable to up to three years imprisonment or a fine | Appropriate range for a regulatory fine:  €275.00 to €500.00  Investigate whether the operator of the abattoir violated Section 130 of the German Act on Regulatory Offences | If an employee has intentionally or negligently caused significant pain or suffering to an animal, an animal protection order should be issued to prohibit the employee from continuing to work in this or any other relevant position, until they have undergone re-training and have provided proof of acquiring the necessary expertise  If the person concerned no longer demonstrates sufficient competence, knowledge, or sense of responsibility to carry out relevant tasks, their certificate of competence according to Article 21 of Regulation (EC) No 1099/2009 may be temporarily or permanently withdrawn  Appropriate range for a regulatory fine:  €550.00 to €1,000.00  File a criminal complaint |  |

**Offense (8):** Intentionally applying pressure to a sensitive body part of an animal, such as the eyes, ears, nose, anus, or genitals, causing undue pain or suffering

| **Citation of legal/regulatory requirements to protect animals at the time of slaughter/killing** | **Citation of relevant regulatory and criminal penalties** | **Special measures/penalties recommended by OV for the first offence** | **Special measures/penalties recommended by OV if the offence is repeated** | **Judicial decisions/ notes** |
| --- | --- | --- | --- | --- |
| Article 15(1) in conjunction with Annex III (1.8.)(b) der Regulation (EC) No 1099/2009 | According to Section 18(3)(2)(a) of the German Animal Welfare Act in conjunction with Section 16(3)(8) of the German Ordinance on the Protection of Animals in Connection with Slaughter or Killing, any person who deliberately or negligently does not ensure that no pressure is applied to a sensitive body part of an animal mentioned in that (in contravention of Article 15(1) in conjunction with Annex III(1.8)(b) shall be guilty of a regulatory offence | Appropriate range for a regulatory fine:  €275.00 to €500.00  Investigate whether the operator of the abattoir violated Section 130 of the German Act on Regulatory Offences | Appropriate range for a regulatory fine:  €550.00 to €1,000.00  If an employee has intentionally or negligently caused significant pain or suffering to an animal, an animal protection order should be issued to prohibit the employee from continuing to work in this or any other relevant position, until they have undergone re-training and have provided proof of acquiring the necessary expertise  If the person concerned no longer demonstrates sufficient competence, knowledge, or sense of responsibility to carry out relevant tasks, their certificate of competence according to Article 21 of Regulation (EC) No 1099/2009 may be temporarily or permanently withdrawn |  |

**Offense (9):** The act of lifting or pulling an animal by its head, fur, ears, horns, legs, or tail

| **Citation of legal/regulatory requirements to protect animals at the time of slaughter/killing** | **Citation of relevant regulatory and criminal penalties** | **Special measures/penalties recommended by OV for the first offence** | **Special measures/penalties recommended by OV if the offence is repeated** | **Judicial decisions/ notes** |
| --- | --- | --- | --- | --- |
| Article 15(1) in conjunction with Annex III(1.8)(c) of Regulation (EC) No 1099/2009 | According to Section 18(3)(2)(a) of the German Animal Welfare Act in conjunction with Section 16(3)(9) German Ordinance on the Protection of Animals in Connection with Slaughter or Killing,  any person who deliberately or negligently fails to ensure that animals are handled in such a way which avoids unnecessary pain or suffering in contravention of Article 15(1) in conjunction with Annex III(1.8)(c) shall be guilty of a regulatory offence | Appropriate range for a regulatory fine:  €275.00 to €500.00  Investigate whether the operator of the abattoir violated Section 130 of the German Act on Regulatory Offences | Appropriate range for a regulatory fine:  €550.00 to €1,000.00  If an employee has intentionally or negligently caused significant pain or suffering to an animal, an animal protection order should be issued to prohibit the employee from continuing to work in this or any other relevant position, until they have undergone re-training and have provided proof of acquiring the necessary expertise  If the person concerned no longer demonstrates sufficient competence, knowledge, or sense of responsibility to carry out relevant tasks, their certificate of competence according to Article 21 of Regulation (EC) No 1099/2009 may be temporarily or permanently withdrawn |  |

**Offense (10):** The act of grabbing, carrying or tugging poultry in contravention of animal welfare regulations (e.g. by only one wing, the neck, head, tail, wing tips or plumage)

| **Citation of legal/regulatory requirements to protect animals at the time of slaughter/killing** | **Citation of relevant regulatory and criminal penalties** | **Special measures/penalties recommended by OV for the first offence** | **Special measures/penalties recommended by OV if the offence is repeated** | **Judicial decisions/ notes** |
| --- | --- | --- | --- | --- |
| Article 15(1) in conjunction with Annex III(1.8)(c) of Regulation (EC) No 1099/2009 | According to Section 18(3)(2)(a) of the German Animal Welfare Act in conjunction with Section 16(3)(9) of the German Ordinance on the Protection of Animals in Connection with Slaughter or Killing, any person who deliberately or negligently does not ensure that an animal is not raised or drawn up on the head, ears, horns, legs, tail or fur in contravention of Article 15(1) in conjunction with Annex III(1.8)(c) shall be guilty of a regulatory offence | Appropriate range for a regulatory fine:  €275.00 to €500.00  Investigate whether the operator of the abattoir violated Section 130 of the German Act on Regulatory Offences | If an employee has intentionally or negligently caused significant pain or suffering to an animal, an animal protection order should be issued to prohibit the employee from continuing to work in this or any other relevant position, until they have undergone re-training and have provided proof of acquiring the necessary expertise  If the person concerned no longer demonstrates sufficient competence, knowledge, or sense of responsibility to carry out relevant tasks, their certificate of competence according to Article 21 of Regulation (EC) No 1099/2009 may be temporarily or permanently withdrawn  Appropriate range for a regulatory fine:  €550.00 to €1,000.00 |  |

**Offense (11):** Forcing animals to move by shouting, or by exhibiting aggressive behavior

| **Citation of legal/regulatory requirements to protect animals at the time of slaughter/killing** | **Citation of relevant regulatory and criminal penalties** | **Special measures/penalties recommended by OV for the first offence** | **Special measures/penalties recommended by OV if the offence is repeated** | **Judicial decisions/ notes** |
| --- | --- | --- | --- | --- |
| Section 3(1) of the German Ordinance on the Protection of Animals in Connection with Slaughter or Killing |  | Instruction/Training on effective, permitted driving aids (on file)  An animal protection order should be issued mandating that animals must be driven in a calm and patient manner, while avoiding causing any avoidable noise | Issue an animal protection order which mandates that unloading and driving animals must be carried out under the supervision of the animal welfare officer  Appropriate framework for a periodic penalty payment:  €150.00 to €450.00  If the person concerned no longer demonstrates sufficient competence, knowledge, or sense of responsibility to carry out relevant tasks, their certificate of competence according to Article 21 of Regulation (EC) No 1099/2009 may be temporarily or permanently withdrawn |  |

**Offense (12):** Improper use of a herding board or rattle paddle

| **Citation of legal/regulatory requirements to protect animals at the time of slaughter/killing** | **Citation of relevant regulatory and criminal penalties** | **Special measures/penalties recommended by OV for the first offence** | **Special measures/penalties recommended by OV if the offence is repeated** | **Judicial decisions/ notes** |
| --- | --- | --- | --- | --- |
| Article 15(1) in conjunction with Annex III(1.8)(c) of Regulation (EC) No 1099/2009 | According to Section 18(3)(2)(a) of the German Animal Welfare Act in conjunction with Section 16(3)(9) German Ordinance on the Protection of Animals in Connection with Slaughter or Killing, any person who deliberately or negligently fails to ensure that animals are handled in such a way which avoids unnecessary pain or suffering in contravention of Article 15(1) in conjunction with Annex III(1.8)(c) shall be guilty of a regulatory offence | Cautionary fine: €55.00  Appropriate range for a regulatory fine:  €150.00 to €450.00  Issue an animal protection order that establishes clear guidelines for the use of herding boards and rattle paddles  Investigate whether the operator of the abattoir violated Section 130 of the German Act on Regulatory Offences | Appropriate range for a regulatory fine/periodic penalty payment:  €300.00 to €900.00 |  |

# Part 3: Measures and Penalties for Inadequate Housing and Husbandry in Lairage

**Offense (13):** Animals are obstructed or prevented from moving in the required direction by obstacles or structural defects, or these issues create the possibility of escape

| **Citation of legal/regulatory requirements to protect animals at the time of slaughter/killing** | **Citation of relevant regulatory and criminal penalties** | **Special measures/penalties recommended by OV for the first offence** | **Special measures/penalties recommended by OV for the event of failure to remedy the defect by the deadline or in the event of repeated offence** | **Judicial decisions/ notes** |
| --- | --- | --- | --- | --- |
| Article 15(1) in conjunction with Annex II(2.1)(a) of Regulation (EC) No 1099/2009 | According to Section 18(3)(2)(a) of the German Animal Welfare Act in conjunction with Section 16(3)(9) German Ordinance on the Protection of Animals in Connection with Slaughter or Killing, any person who deliberately or negligently fails to ensure that animals are handled in such a way which avoids unnecessary pain or suffering in contravention of Article 15(1) in conjunction with Annex III(1.8)(c) shall be guilty of a regulatory offence | Issue an animal protection order that outlines specific corrective actions that must be taken to ensure the well-being of the animals  Investigate whether the operator of the abattoir violated Section 130 of the German Act on Regulatory Offences | Appropriate framework for a periodic penalty payment:  €150.00 to €450.00 |  |

**Offense (14):** The permanent unevenness of the ground, such as holes, can compromise the surefootedness of animals walking on floors (e.g. passageways and holding pens)

| **Citation of legal/regulatory requirements to protect animals at the time of slaughter/killing** | **Citation of relevant regulatory and criminal penalties** | **Special measures/penalties recommended by OV for the first offence** | **Special measures/penalties recommended by OV for the event of failure to remedy the defect by the deadline or in the event of repeated offence** | **Judicial decisions/ notes** |
| --- | --- | --- | --- | --- |
| Section 6(2) of the German Ordinance on the Protection of Animals in Connection with Slaughter or Killing | According to Section 18(1)(1)(a) of the German Animal Welfare Act in conjunction with Section 16(1)(2) of the German Ordinance on the Protection of Animals in Connection with Slaughter or Killing, any person who deliberately or negligently does not ensure that the ground is non-slip in contravention of Section 6(2) shall be guilty of a regulatory offence | If necessary, close off the area with defects  Issue an animal protection order that mandates the prompt remediation of any structural defects that may pose a threat to the well-being of the animals  Investigate whether the operator of the abattoir violated Section 130 of the German Act on Regulatory Offences | Regulatory offence: The fine depends on the risk of injury for the animals |  |

**Offense (15):** Animals do not have access to an adequate supply of drinking water

| **Citation of legal/regulatory requirements to protect animals at the time of slaughter/killing** | **Citation of relevant regulatory and criminal penalties** | **Special measures/penalties recommended by OV for the first offence** | **Special measures/penalties recommended by OV for the event of failure to remedy the defect by the deadline or in the event of repeated offence** | **Judicial decisions/ notes** |
| --- | --- | --- | --- | --- |
| Section 7(2)(1) of the German Ordinance on the Protection of Animals in Connection with Slaughter or Killing | According to Section 18(3)(2)(a) of the German Animal Welfare Act in conjunction with Section 16(3)(9) of the German Ordinance on the Protection of Animals in Connection with Slaughter or Killing, any person who deliberately or negligently fails to ensure that animals are handled in such a way which avoids unnecessary pain or suffering in contravention of Article 15(1) in conjunction with Annex III(1.8)(c) shall be guilty of a regulatory offence | Issue an animal protection order that sets a deadline for the remediation of any defects, and consider implementing measures such as limiting the number of animals in pens, requiring responsible employees to attend a seminar, or arranging for a different delivery or slaughter schedule  Framework of fines:  €360.00 to €950.00  Investigate whether the operator of the abattoir violated Section 130 of the German Act on Regulatory Offences | Appropriate range for a regulatory fine/periodic penalty payment:  €720.00 to €1,900.00 |  |

**Offense (16):** Animals arriving at the abattoir in containers (e.g. poultry) are not provided with drinking water despite not being sent to slaughter within two hours upon arrival

| **Citation of legal/regulatory requirements to protect animals at the time of slaughter/killing** | **Citation of relevant regulatory and criminal penalties** | **Special measures/penalties recommended by OV for the first offence** | **Special measures/penalties recommended by OV for the event of failure to remedy the defect by the deadline or in the event of repeated offence** | **Judicial decisions/ notes** |
| --- | --- | --- | --- | --- |
| Section 7(2)(2) of the German Ordinance on the Protection of Animals in Connection with Slaughter or Killing  Section 5(2)(3) of the German Ordinance on the Protection of Animals in Connection with Slaughter or Killing | According to Section 18(1)(3)(a) of the German Animal Welfare Act in conjunction with Section 16(1)(4) German Ordinance on the Protection of Animals in Connection with Slaughter or Killing,  any person who deliberately or negligently fails to supply water in contravention of Section 7(2)(2) shall be guilty of a regulatory offence | Investigate and rectify the cause if possible, e.g. by restructuring the sequence of operations  Issue an animal protection order that requires the abattoir to submit a plan outlining how they will rectify the underlying problem, addressing logistics, personnel, and/or construction as needed  Appropriate framework of fines:  €500.00 to €1,000.00  Investigate whether the operator of the abattoir violated Section 130 of the German Act on Regulatory Offences | Appropriate range for a regulatory fine/periodic penalty payment:  €1,000.00 to €2,000.00 | The following violation resulted in the imposition of a regulatory fine:  For a period of 8 hours, around 750 turkeys were confined in a delivery vehicle without access to water.  Source: Administrative court Oldenburg,  Judgment of 21.10.2015,  Reference number: 11 A 3678/14  Note: Consider the total time the animals remained without water supply (transport and time after arriving at the abattoir) as well as the conditions in the holding area (e.g. fans/roofing/heat). |

**Offense (17):** Animals not slaughtered within six hours after arriving at the abattoir are not provided with appropriate or sufficient feed, or there are not enough troughs, or there isn’t a sufficient trough length per animal to ensure access to feed

| **Citation of legal/regulatory requirements to protect animals at the time of slaughter/killing** | **Citation of relevant regulatory and criminal penalties** | **Special measures/penalties recommended by OV for the first offence** | **Special measures/penalties recommended by OV for the event of failure to remedy the defect by the deadline or in the event of repeated offence** | **Judicial decisions/ notes** |
| --- | --- | --- | --- | --- |
| Section 7(3) of the German Ordinance on the Protection of Animals in Connection with Slaughter or Killing | According to Section 18(3)(2)(a) of the German Animal Welfare Act in conjunction with Section 16(3)(9) German Ordinance on the Protection of Animals in Connection with Slaughter or Killing, any person who deliberately or negligently fails to ensure that animals are handled in such a way which avoids unnecessary pain or suffering in contravention of Article 15(1) in conjunction with Annex III(1.8)(c) shall be guilty of a regulatory offence | Issue an animal protection order requiring the rectification of the identified issue or deficiency, including measures such as limiting the number of animals in pens, reorganizing delivery or slaughter times if necessary, using mobile troughs for feeding if needed, and inspecting feed supplies and relevant checklists  If the responsible employee is found to be liable, order them to undergo follow-up training to prevent future violations  Investigate whether the operator of the abattoir violated Section 130 of the German Act on Regulatory Offences | Appropriate range for a regulatory fine/periodic penalty payment:  €360.00 to €915.00  Investigate whether the operator of the abattoir violated Section 130 of the German Act on Regulatory Offences |  |

**Offense (18):** The holding pens are overcrowded, thereby not providing enough space for every animal to lie down or stand up without hindrance

| **Citation of legal/regulatory requirements to protect animals at the time of slaughter/killing** | **Citation of relevant regulatory and criminal penalties** | **Special measures/penalties recommended by OV for the first offence** | **Special measures/penalties recommended by OV for the event of failure to remedy the defect by the deadline or in the event of repeated offence** | **Judicial decisions/ notes** |
| --- | --- | --- | --- | --- |
| Section 8(2)(1) of the German Ordinance on the Protection of Animals in Connection with Slaughter or Killing | According to Section 18(3)(2)(a) of the German Animal Welfare Act in conjunction with Section 16(3)(9) German Ordinance on the Protection of Animals in Connection with Slaughter or Killing, any person who deliberately or negligently fails to ensure that animals are handled in such a way which avoids unnecessary pain or suffering in contravention of Article 15(1) in conjunction with Annex III(1.8)(c) shall be guilty of a regulatory offence | Issue an animal protection order that requires compliance with maximum occupancy limits for holding pens  Verify that the EU approval includes the appropriate size and occupancy limits for holding pens at the time of approval  Investigate the cause of the issue, including a review of the standard operating procedures (SOPs) and conducting an official hearing with those involved. Based on the findings, identify and implement appropriate improvements, such as specifying the maximum number of animals allowed per pen  Appropriate range for a regulatory fine:  €250.00 to €750.00  Investigate whether the operator of the abattoir violated Section 130 of the German Act on Regulatory Offences | Appropriate range for a regulatory fine/periodic penalty payment:  €500.00 to €1,500.00 | According to the survey conducted during the preparation of this catalog, regulatory fines of €450.00 and  €500.00 were imposed in response to this violation |

**Offense (19):** The holding area or unloading area is not properly weather-proofed, resulting in animals being exposed to adverse weather conditions

| **Citation of legal/regulatory requirements to protect animals at the time of slaughter/killing** | **Citation of relevant regulatory and criminal penalties** | **Special measures/penalties recommended by OV for the first offence** | **Special measures/penalties recommended by OV for the event of failure to remedy the defect by the deadline or in the event of repeated offence** | **Judicial decisions/ notes** |
| --- | --- | --- | --- | --- |
| Section (1)(2) of the German Animal Welfare Act in conjunction with Article 14 Annex II(2.6) of Regulation (EC) No 1099/2009 | According to Section 18(3)(2)(a) of the German Animal Welfare Act in conjunction with Section 16(3)(9) German Ordinance on the Protection of Animals in Connection with Slaughter or Killing, any person who deliberately or negligently fails to ensure that animals are handled in such a way which avoids unnecessary pain or suffering in contravention of Article 15(1) in conjunction with Annex III(1.8)(c) shall be guilty of a regulatory offence | Issue an animal protection order that requires the rectification of any identified deficiencies and the implementation of measures to ensure adequate housing conditions  Investigate whether the operator of the abattoir violated Section 130 of the German Act on Regulatory Offences | Appropriate range for a regulatory fine/periodic penalty payment:  €150.00 to €450.00 | Note: The proposal involving instructions to rectify the defect must also take difficult weather conditions into account |

**Offense (20):** Failure to provide sufficient bedding or any bedding in the holding area

| **Citation of legal/regulatory requirements to protect animals at the time of slaughter/killing** | **Citation of relevant regulatory and criminal penalties** | **Special measures/penalties recommended by OV for the first offence** | **Special measures/penalties recommended by OV for the event of failure to remedy the defect by the deadline or in the event of repeated offence** | **Judicial decisions/ notes** |
| --- | --- | --- | --- | --- |
| Section (1)(2) of the German Animal Welfare Act  in conjunction with  Article 15(1) mit Annex III (1.2.) Regulation (EC) No 1099/2009 in conjunction with Section 8 (2 (2 of the German Ordinance on the Protection of Animals in Connection with Slaughter or Killing | According to Section 18(3)(2)(a) of the German Animal Welfare Act in conjunction with Section 16(3)(9) of the German Ordinance on the Protection of Animals in Connection with Slaughter or Killing, any person who deliberately or negligently fails to ensure that animals are handled in such a way which avoids unnecessary pain or suffering in contravention of Article 15(1) in conjunction with Annex III(1.8)(c) shall be guilty of a regulatory offence | Issue an animal protection order that requires providing adequate bedding for animals in the holding area, and developing an emergency plan that ensures sufficient quantities of bedding are available at all times  Investigate whether the operator of the abattoir violated Section 130 of the German Act on Regulatory Offences | Appropriate range for a regulatory fine/periodic penalty payment:  €250.00 to €750.00 | Note: Section (1)(2) of the German Animal Welfare Act does not apply if the animals which leave the holding pens within six hours after their arrival at the abattoir to be slaughtered |

**Offense (21):** Deliberately throwing, dropping or knocking over a container with live animals

| **Citation of legal/regulatory requirements to protect animals at the time of slaughter/killing** | **Citation of relevant regulatory and criminal penalties** | **Special measures/penalties recommended by OV for the first offence** | **Special measures/penalties recommended by OV if the offence is repeated** | **Judicial decisions/ notes** |
| --- | --- | --- | --- | --- |
| Article 15(1) in conjunction with Annex III (1.3.)(1)(a) of Regulation (EC) No 1099/2009 | According to Section 18(3)(2)(a) of the German Animal Welfare Act in conjunction with Section 16(1)(4) German Ordinance on the Protection of Animals in Connection with Slaughter or Killing, any person who deliberately or negligently does not ensure that a transport container with animals is not thrown, dropped or knocked over in contravention of Article 15(1) in conjunction with Annex III (1.3.)(1) (a) shall be guilty of a regulatory offence | Appropriate range for a regulatory fine:  €275.00 to €550.00  Investigate whether the operator of the abattoir violated Section 130 of the German Act on Regulatory Offences | Appropriate range for a regulatory fine:  €550.00 to €1,100.00  If an employee has intentionally or negligently caused significant pain or suffering to an animal, an animal protection order should be issued to prohibit the employee from continuing to work in this or any other relevant position, until they have undergone re-training and have provided proof of acquiring the necessary expertise  If the person concerned no longer demonstrates sufficient competence, knowledge, or sense of responsibility to carry out relevant tasks, their certificate of competence according to Article 21 of Regulation (EC) No 1099/2009 may be temporarily or permanently withdrawn |  |

**Offense (22):** Animals of different species, sexes, ages, or origins are housed together (despite clearly being incompatible) which may lead to fights, injuries, and unnecessary stress

| **Citation of legal/regulatory requirements to protect animals at the time of slaughter/killing** | **Citation of relevant regulatory and criminal penalties** | **Special measures/penalties recommended by OV for the first offence** | **Special measures/penalties recommended by OV for the event of failure to remedy the defect by the deadline or in the event of repeated offence** | **Judicial decisions/ notes** |
| --- | --- | --- | --- | --- |
| Section 7(4) of the German Ordinance on the Protection of Animals in Connection with Slaughter or Killing | According to Section 18(3)(2)(a) of the German Animal Welfare Act in conjunction with Section 16(3)(9) of the German Ordinance on the Protection of Animals in Connection with Slaughter or Killing, any person who deliberately or negligently fails to ensure that animals are handled in such a way which avoids unnecessary pain or suffering in contravention of Article 15(1) in conjunction with Annex III(1.8)(c) shall be guilty of a regulatory offence | Issue an animal protection order that requires animals to be separated by species, sex, and origin, and requests the abattoir to submit a concept for addressing the problem in terms of logistics, personnel, and/or construction. If necessary, order the use of mobile separators  Investigate whether the animals were transported together and, if so, schedule an official hearing with the livestock driver, and subsequently mandate measures if necessary  Appropriate range for a regulatory fine:  €150.00 to €550.00  Investigate whether the operator of the abattoir violated Section 130 of the German Act on Regulatory Offences | Regulatory offence: Appropriate range for a regulatory fine/periodic penalty payment:  €300.00 to €1,100.00 | According to the survey conducted within the preparation of this catalog, housing bulls and female cattle together over night was sanctioned with a regulatory fine of €500.00 |

**Offense (23):** Tying an animal's legs together or to a post in violation of animal welfare standards

| **Citation of legal/regulatory requirements to protect animals at the time of slaughter/killing** | **Citation of relevant regulatory and criminal penalties** | **Special measures/penalties recommended by OV for the first offence** | **Special measures/penalties recommended by OV if the offence is repeated** | **Judicial decisions/ notes** |
| --- | --- | --- | --- | --- |
| Article 15(1) in conjunction with Annex III(1.10)(1) of Regulation (EC) No 1099/2009 | According to Section 18(3)(2)(a) of the German Animal Welfare Act in conjunction with Section 16(3)(14) of the German Ordinance on the Protection of Animals in Connection with Slaughter or Killing, any person who deliberately or negligently does not ensure that an animal is not tied in the manner specified therein or that its legs are not tied together in contravention of Article 15(1) in conjunction with Annex III(1.10)(1), shall be guilty of a regulatory offence  According to Section 17(2)(a) and/or (b) of the German Animal Welfare Act, any person who commits an offence out of cruelty or if the animal was subjected to prolonged or repeated significant pain or suffering, shall be liable to up to three years imprisonment or a fine | Appropriate range for a regulatory fine:  €300.00 to €1,500.00  Investigate whether the operator of the abattoir violated Section 130 of the German Act on Regulatory Offences  File a criminal complaint | Appropriate range for a regulatory fine:  €600.00 to €3,000.00  If the person concerned no longer demonstrates sufficient competence, knowledge, or sense of responsibility to carry out relevant tasks, their certificate of competence according to Article 21 of Regulation (EC) No 1099/2009 may be temporarily or permanently withdrawn  File a criminal complaint | The following violation resulted in the imposition of a regulatory fine: Tying up the limbs of a sheep to prevent it from jumping; transporting it to the abattoir in this position and leaving it in this state overnight led to a of €500.00.  Source: A document containing regulatory fines issued by the administrative district of Kassel (reporting year: 2019) |

# Part 4: Measures and Penalties for Violations Related to Inappropriate Handling of Animals with Special Care Needs

**Offense (24):** Lactating dairy cattle are not milked at least every twelve hours

| **Citation of legal/regulatory requirements to protect animals at the time of slaughter/killing** | **Citation of relevant regulatory and criminal penalties** | **Special measures/penalties recommended by OV for the first offence** | **Special measures/penalties recommended by OV if the offence is repeated** | **Judicial decisions/ notes** |
| --- | --- | --- | --- | --- |
| Annex III(1.5)(2)(a) of Regulation (EC) No 1099/2009 | According to Section 18(3)(2)(a) of the German Animal Welfare Act in conjunction with Section 16(3)(5) German Ordinance on the Protection of Animals in Connection with Slaughter or Killing,  any person who deliberately or negligently does not ensure that lactating dairy cattle is milked at least every twelve hours in contravention of Article 15(1) in conjunction with Annex III(1.5)(2)(a), shall be guilty of a regulatory offence  According to Section 17(2)(a) and/or (b) of the German Animal Welfare Act, any person who commits an offence out of cruelty or if the animal was subjected to prolonged or repeated significant pain or suffering, shall be liable to up to three years imprisonment or a fine | If applicable, train employees to recognize special care need  Appropriate range for a regulatory fine: €300.00 to €1,500.00 pro Tier  Investigate whether the operator of the abattoir violated Section 130 of the German Act on Regulatory Offences | Appropriate range for a regulatory fine:  €600.00 to €3,000.00 pro Tier  File a criminal complaint | The following violation resulted in the imposition of a regulatory fine: The vast majority of animals (128) consisted of visibly lactating cows which had not been milked. This offense, together with numerous other violations of hygiene and animal welfare regulations, was sanctioned with a large fine.  Source: Kiel Local Court,  Court order from September 28^th^, 2018,  Reference number: 590 Js 10044/16 |

**Offense (25):** Dragging animals that are too weak or injured to walk on their own using painful tools such as a winch or other driving aids

| **Citation of legal/regulatory requirements to protect animals at the time of slaughter/killing** | **Citation of relevant regulatory and criminal penalties** | **Special measures/penalties recommended by OV for the first offence** | **Special measures/penalties recommended by OV for the event of failure to remedy the defect by the deadline or in the event of repeated offence** | **Judicial decisions/ notes** |
| --- | --- | --- | --- | --- |
| Section 8(1) of the German Ordinance on the Protection of Animals in Connection with Slaughter or Killing | According to Section 18(1)(3)(b) of the German Animal Welfare Act in conjunction with Section 16(2)(1) and (2) of the German Ordinance on the Protection of Animals in Connection with Slaughter or Killing, any person who deliberately or negligently does not ensure that an animal named therein is slaughtered or killed in contravention of Section 8(1)(1)(1) or (2) shall be guilty of a regulatory offence | Slaughter/kill the animal on site  Issue an animal protection order that mandates the abattoir to extend its standard operating procedure for handling diseased or injured animals, and to conduct inspections upon delivery  Clarify if the animal was already unfit for transport before it was transported to the abattoir. Further measures are necessary if this is the case.  Appropriate range for a regulatory fine: €500.00 to €1,500.00  Investigate whether the operator of the abattoir violated Section 130 of the German Act on Regulatory Offences  File a criminal complaint | Appropriate range for a regulatory fine/periodic penalty payment:  €1,000.00 to €3,000.00  File a criminal complaint | Punished as criminal offences in past cases: 1) An injured cow was pulled from a truck using a winch and subsequently suspended by one hind leg, during which time it displayed signs of struggling. The cow was only stunned once it was being hoisted up.  A fine of 40 daily rates was imposed for this offense.  Source: Stendal District Court,  Penalty order,  Reference number: Cs 444 Js 15746/18  2) An injured cow was pulled from a truck using a winch, and only stunned after being pulled into the abattoir. While being pulled off the truck, the cow displayed defensive movements with its neck and head, attesting to the significant pain it was suffering.  A fine of 40 daily rates was imposed for this offense.  Source: Local court Stendal,  Penalty order from June 22^nd^, 2020,  Reference number: Cs 444 Js 17063/18  3) Three injured cows were pulled from a truck and pulled to the entrance area of the abattoir using a winch  A fine of 100 daily rates was imposed for this offense.  Source: Bad Iburg Local Court,  Court verdict from November 2nd, 2020,  Reference number: 23 Ds (1102 Js 23602/20) 282/20 |

| **Citation of legal/regulatory requirements to protect animals at the time of slaughter/killing** | **Citation of relevant regulatory and criminal penalties** | **Special measures/penalties recommended by OV for the first offence** | **Special measures/penalties recommended by OV for the event of failure to remedy the defect by the deadline or in the event of repeated offence** | **Judicial decisions/ notes** |
| --- | --- | --- | --- | --- |
|  | According to Section 18(1)(3)(b) of the German Animal Welfare Act in conjunction with Section 16(2)(1) and (2) of the German Ordinance on the Protection of Animals in Connection with Slaughter or Killing, any person who deliberately or negligently does not ensure that an animal mentioned therein is stunned or killed in contravention of Section 8(1)(1)(3) shall be guilty of a regulatory offence  According to Section 17(2)(a) and/or (b) of the German Animal Welfare Act, any person who commits an offence out of cruelty or if the animal was subjected to prolonged or repeated significant pain or suffering, shall be liable to up to three years imprisonment or a fine |  |  |  |

**Offense (26):** Sick or injured animals (which are obviously in severe pain, have large or deep wounds, are bleeding severely, or show a severely disturbed general condition) are housed with healthy animals in holding pens instead of being prioritised for immediate slaughter or euthanasia

| **Citation of legal/regulatory requirements to protect animals at the time of slaughter/killing** | **Citation of relevant regulatory and criminal penalties** | **Special measures/penalties recommended by OV for the first offence** | **Special measures/penalties recommended by OV if the offence is repeated** | **Judicial decisions/ notes** |
| --- | --- | --- | --- | --- |
| Section 8(1)(1)(1) and (2) of the German Ordinance on the Protection of Animals in Connection with Slaughter or Killing | According to Section 18(1)(3)(b) of the German Animal Welfare Act in conjunction with Section 16(2)(1) and (2) of the German Ordinance on the Protection of Animals in Connection with Slaughter or Killing, any person who deliberately or negligently does not ensure that an animal named therein is not slaughtered or killed in contravention of Section 8(1)(1)(1) or (2) shall be guilty of a regulatory offence | Issue an animal protection order that mandates the immediate on-site slaughter or killing of sick or injured animals. Furthermore, order the abattoir to expand its standard operating procedure for handling such animals and to conduct inspections upon animal delivery. If necessary, also order permanent monitoring upon arrival of the animals  Investigate whether the animal was already unfit for transport prior to being transported; and, if this was the case, order further measures  Investigate whether the operator of the abattoir violated Section 130 of the German Act on Regulatory Offences  Appropriate range for a regulatory fine:  €250.00 to €750.00 | If the person concerned no longer demonstrates sufficient competence, knowledge, or sense of responsibility to carry out relevant tasks, their certificate of competence according to Article 21 of Regulation (EC) No 1099/2009 may be temporarily or permanently withdrawn  Appropriate range for a regulatory fine/periodic penalty payment:  €500.00 to €1,500.00 |  |

# Part 5: Measures and Penalties for Improper Restraint, Stunning, and Bleeding of Animals at Abattoirs

**Offense (27):** Inadequate immobilisation of an animal prior to stunning (e.g. the animal can turn around in the stun box/no head restraint when immobilising solipeds or cattle)

| **Citation of legal/regulatory requirements to protect animals at the time of slaughter/killing** | **Citation of relevant regulatory and criminal penalties** | **Special measures/penalties recommended by OV for the first offence** | **Special measures/penalties recommended by OV for the event of failure to remedy the defect by the deadline or in the event of repeated offence** | **Judicial decisions/ notes** |
| --- | --- | --- | --- | --- |
| Section 11 of the German Ordinance on the Protection of Animals in Connection with Slaughter or Killing | According to Section 18(3)(2)(a) of the German Animal Welfare Act in conjunction with Section 16(3)(9) of the German Ordinance on the Protection of Animals in Connection with Slaughter or Killing, any person who deliberately or negligently fails to ensure that animals are handled in such a way which avoids unnecessary pain or suffering in contravention of Article 15(1) in conjunction with Annex III(1.8)(c) shall be guilty of a regulatory offence | Issue an animal protection order that mandates the adequate restraint of animals and prohibits slaughter under the current structural conditions. Additionally, order the abattoir to remedy any logistical or constructional problems that may be causing inadequate restraint  Appropriate range for a regulatory fine:  €200.00 to €600.00  Investigate whether the operator of the abattoir violated Section 130 of the German Act on Regulatory Offences | Appropriate range for a regulatory fine/periodic penalty payment:  €400.00 to €1,200.00 | Note: Section 11(1)(3) of the German Ordinance on the Protection of Animals in Connection with Slaughter or Killing states that pigs weighing more than 30 kilograms must be individually immobilised in stunning traps or similar devices when electric stunning is applied at abattoirs where pigs are slaughtered on a scale which, according to the conversion rate laid down in Article 17(6)(c) and (d) of Regulation (EC) No 1099/2009 amounts to more than 20 livestock units per week or more than 1000 livestock units per year. This regulation violates Article 3 (1) of Regulation (EC) 1099/2009 and Article 3 (1) of the German Basic Law and may therefore not be applied in the context of an animal welfare order based on Section 16a (1)(1) of the German Animal Welfare Act.  Source: Administrative court Karlsruhe, judgment of 14.03.2019,  Reference number:12 K 3450/16 |

**Offense (28):** Prohibited methods of immobilizing animals, such as using a bolt shot to the neck

| **Citation of legal/regulatory requirements to protect animals at the time of slaughter/killing** | **Citation of relevant regulatory and criminal penalties** | **Special measures/penalties recommended by OV for the first offence** | **Special measures/penalties recommended by OV for the event of failure to remedy the defect by the deadline or in the event of repeated offence** | **Judicial decisions/ notes** |
| --- | --- | --- | --- | --- |
| Article 15(3)(1) of Regulation (EC) No 1099/2009 | According to Section 18(3)(2)(b) of the German Animal Welfare Act in conjunction with Section 16(4)(10) of the German Ordinance on the Protection of Animals in Connection with Slaughter or Killing, any person who deliberately or negligently uses a method to immobilize animals in contravention of Article 15(3)(1) shall be guilty of a regulatory offence  According to Section 17(2)(a) and/or (b) of the German Animal Welfare Act, any person who commits an offence out of cruelty or if the animal was subjected to prolonged or repeated significant pain or suffering, shall be liable to up to three years imprisonment or a fine | Develop/review emergency plan  If necessary, issue an animal protection order to rectify the underlying issue, depending on the cause  Investigate whether the operator of the abattoir violated Section 130 of the German Act on Regulatory Offences  Appropriate range for a regulatory fine:  €300.00 to €800.00  File a criminal complaint | Appropriate range for a regulatory fine/periodic penalty payment:  €600.00 to €1,600.00  If the person concerned no longer demonstrates sufficient competence, knowledge, or sense of responsibility to carry out relevant tasks, their certificate of competence according to Article 21 of Regulation (EC) No 1099/2009 may be temporarily or permanently withdrawn  File a criminal complaint | Note: Violations of this kind often occur when structural deficiencies prevent the proper immobilization of animals for stunning. In such cases, addressing the deficiencies is crucial. However, intentional acts of animal mistreatment that do not involve structural impediments should be considered regulatory or criminal offenses. |

**Offense (29):** Using a stunning device with visible defects (such as corroded electrodes, a bent bolt, or worn buffer rubbers) to stun an animal

| **Citation of legal/regulatory requirements to protect animals at the time of slaughter/killing** | **Citation of relevant regulatory and criminal penalties** | **Special measures/penalties recommended by OV for the first offence** | **Special measures/penalties recommended by OV if the offence is repeated** | **Judicial decisions/ notes** |
| --- | --- | --- | --- | --- |
| Article 9(1)(1) of Regulation (EC) No 1099/2009 | According to Section 18(3)(2)(b) of the German Animal Welfare Act in conjunction with Section 16(4)(2) of the German Ordinance on the Protection of Animals in Connection with Slaughter or Killing,  any person who deliberately or negligently does not ensure that any equipment referred to therein is maintained or inspected in contravention of Article 9(1)(1) shall be guilty of a regulatory offence | Issue an animal protection order mandating that slaughter be halted until a replacement or repaired device is ready, and that the device must be inspected before use. Only intact and inspected equipment may be used. Additionally, require the responsible employee to attend a seminar in case of any employee fault  Order a cautionary fine  Investigate whether the operator of the abattoir violated Section 130 of the German Act on Regulatory Offences  Appropriate range for a regulatory fine:  €250.00 to €600.00 | Appropriate range for a regulatory fine/periodic penalty payment:  €500.00 to €1,200.00 |  |

**Offense (30):** The absence of proper spare equipment or replacement parts for worn components of bolt gun equipment (e.g. buffer rubbers, recuperating spring) during stunning

| **Citation of legal/regulatory requirements to protect animals at the time of slaughter/killing** | **Citation of relevant regulatory and criminal penalties** | **Special measures/penalties recommended by OV for the first offence** | **Special measures/penalties recommended by OV if the offence is repeated** | **Judicial decisions/ notes** |
| --- | --- | --- | --- | --- |
| Article 9(2) of Regulation (EC) No 1099/2009 | According to Section 18(3)(2)(b) of the German Animal Welfare Act in conjunction with  Section 16(4)(2) of the German Ordinance on the Protection of Animals in Connection with Slaughter or Killing, any person who deliberately or negligently does not ensure that a device referred to therein is maintained or inspected in contravention of Article 9(1)(1) shall be guilty of a regulatory offence | Issue an animal protection order mandating the immediate suspension of slaughter until the necessary spare parts are made available on-site  Appropriate range for a regulatory fine:  €250.00 to €600.00  Investigate whether the operator of the abattoir violated Section 130 of the German Act on Regulatory Offences | Appropriate range for a regulatory fine/periodic penalty payment:  €500.00 to €1,200.00 |  |

**Offense (31):** The use of outdated or old stunning equipment that does not meet current animal welfare standards

| **Citation of legal/regulatory requirements to protect animals at the time of slaughter/killing** | **Citation of relevant regulatory and criminal penalties** | **Special measures/penalties recommended by OV for the first offence** | **Special measures/penalties recommended by OV for the event of failure to remedy the defect by the deadline or in the event of repeated offence** | **Judicial decisions/ notes** |
| --- | --- | --- | --- | --- |
| Article 9(1)(1) of Regulation (EC) No 1099/2009 | According to Section 18(3)(2)(b) of the German Animal Welfare Act in conjunction with Section 16(4)(2) of the German Ordinance on the Protection of Animals in Connection with Slaughter or Killing, any person who deliberately or negligently does not ensure that any equipment referred to therein is maintained or inspected in contravention of Article 9 (1)(1) shall be guilty of a regulatory offence | Order an immediate halt to the slaughter process and mandate that it may only resume once new equipment is operational and available on site, with the stipulation that stunning must be carried out exclusively using the new equipment  Appropriate range for a regulatory fine:  €250.00 to €600.00 and skim excess profit according to Section 10 of the Federal Act Against Unfair Competition  Investigate whether the operator of the abattoir violated Section 130 of the German Act on Regulatory Offences | Appropriate range for a regulatory fine/periodic penalty payment:  €500.00 to €1,200.00 |  |

**Offense (32):** The water bath stunning equipment is inadequate

| **Citation of legal/regulatory requirements to protect animals at the time of slaughter/killing** | **Citation of relevant regulatory and criminal penalties** | **Special measures/penalties recommended by OV for the first offence** | **Special measures/penalties recommended by OV if the offence is repeated** | **Judicial decisions/ notes** |
| --- | --- | --- | --- | --- |
| Article 14(1) in conjunction with Annex II(5.10)(1) of Regulation (EC) No 1099/2009 | According to Section 18(3)(2)(b) of the German Animal Welfare Act in conjunction with Section 16 (4 (6 of the German Ordinance on the Protection of Animals in Connection with Slaughter or Killing, any person who deliberately or negligently does not ensure that the water basin is laid out or maintained in the manner the manner prescribed therein in contravention of Article 14(1) in conjunction with Annex II (5.7)(2) shall be guilty of a regulatory offence  According to Section 17(2)(a) and/or (b) of the German Animal Welfare Act, any person who commits an offence out of cruelty or if the animal was subjected to prolonged or repeated significant pain or suffering, shall be liable to up to three years imprisonment or a fine | Issue an animal protection order mandating an immediate interruption of slaughter and requiring the abattoir to ensure that the water basin is properly designed and maintained according to regulations  Appropriate range for a regulatory fine:  €250.00 to €600.00  Investigate whether the operator of the abattoir violated Section 130 of the German Act on Regulatory Offences  File a criminal complaint | Issue an animal protection order which mandates the immediate interruption of slaughtering  Appropriate range for a regulatory fine/periodic penalty payment:  €500.00 to €1,200.00  File a criminal complaint |  |

**Offense (33):** The attachment of the stunning device is incorrect (e.g. the bolt firing device is not positioned on the head correctly, such as not being vertical or secure, causing the bolt to be fired incorrectly or not making contact)

| **Citation of legal/regulatory requirements to protect animals at the time of slaughter/killing** | **Citation of relevant regulatory and criminal penalties** | **Special measures/penalties recommended by OV for the first offence** | **Special measures/penalties recommended by OV if the offence is repeated** | **Judicial decisions/ notes** |
| --- | --- | --- | --- | --- |
| Section 16(2)(3) of the German Ordinance on the Protection of Animals in Connection with Slaughter or Killing | According to Section 18(1)(3)(b) of the German Animal Welfare Act in conjunction with Section 16(2)(3) of the German Ordinance on the Protection of Animals in Connection with Slaughter or Killing, any person who deliberately or negligently kills a vertebrate in contravention of Section 12(3)(1) shall be guilty of a regulatory offence | Issue an animal protection order mandating the immediate interruption of slaughter, and prohibit employees involved in cases where significant pain or suffering has been intentionally or negligently inflicted on an animal. Re-training and proof of expertise acquisition may be required before allowing such employees to resume work  Investigate whether the operator of the abattoir violated Section 130 of the German Act on Regulatory Offences:  Verification if persons responsible for operations are contributing to the root problem (e.g. structural deficiencies and/or fixation not in place).  Appropriate range for a regulatory fine:  €300.00 to €1,000.00 | If the person concerned no longer demonstrates sufficient competence, knowledge, or sense of responsibility to carry out relevant tasks, their certificate of competence according to Article 21 of Regulation (EC) No 1099/2009 may be temporarily or permanently withdrawn  Appropriate range for a regulatory fine:  €600.00 to €2,000.00 |  |

**Offense (34):** Failure to perform an assessment of the effectiveness of stunning

| **Citation of legal/regulatory requirements to protect animals at the time of slaughter/killing** | **Citation of relevant regulatory and criminal penalties** | **Special measures/penalties recommended by OV for the first offence** | **Special measures/penalties recommended by OV if the offence is repeated** | **Judicial decisions/ notes** |
| --- | --- | --- | --- | --- |
| Section 4 of the German Animal Welfare Act;  Section 3 Paragraph  1 of the German Ordinance on the Protection of Animals in Connection with Slaughter or Killing | According to Section 18(1)(5) of the German Animal Welfare Act, any person who deliberately or negligently kills a vertebrate in contravention of Section 4 (1, shall be guilty of a regulatory offence) | Issue an animal protection order mandating the monitoring of stunning effectiveness. Require employees to work under the supervision of an experienced person with a certificate of competence for a specific period, such as three months  Investigate whether the operator of the abattoir violated Section 130 of the German Act on Regulatory Offences  Appropriate range for a regulatory fine:  €300.00 to €1,000.00 | Appropriate range for a regulatory fine:  €600.00 to €2,000.00 |  |

**Offense (35):** An animal is slaughtered without prior stunning, without a respective official exemption permit (e.g. for the purpose of Halal/Kosher slaughter)

| **Citation of legal/regulatory requirements to protect animals at the time of slaughter/killing** | **Citation of relevant regulatory and criminal penalties** | **Special measures/penalties recommended by OV for the first offence** | **Special measures/penalties recommended by OV if the offence is repeated** | **Judicial decisions/ notes** |
| --- | --- | --- | --- | --- |
| Section 4a(2)(2) of the German Animal Welfare Act | According to Section 18(1)(6) of the German Animal Welfare Act, any person who deliberately or negligently kills a warm-blooded animal in contravention of Section 4a(1) shall be guilty of a regulatory offence  According to Section 17(2)(a) and/or (b) of the German Animal Welfare Act, any person who commits an offence out of cruelty or if the animal was subjected to prolonged or repeated significant pain or suffering, shall be liable to up to three years imprisonment or a fine | Issue an animal protection order which mandates the immediate arrest of slaughter  Contact the licensing authority. Prohibition of business due to unreliability  Specifically train employees on appropriate stunning methods  Appropriate range for a regulatory fine:  €500.00 to €1,000.00  File a criminal complaint | Issue an animal protection order which mandates the immediate arrest of slaughter  Contact the licensing authority. Prohibition of business due to unreliability  Appropriate range for a regulatory fine:  €1,000.00 to €2,000.00  File a criminal complaint | Note: If necessary, confiscate the carcass and arrange for a post-mortem examination to confirm whether the animal was slaughtered without stunning |

**Offense (36):** An animal that has been ineffectively stunned (e.g., one that shows signs of consciousness such as spontaneous blinking, directed eye movements or reactions to touch) is not re-stunned before exsanguination

| **Citation of legal/regulatory requirements to protect animals at the time of slaughter/killing** | **Citation of relevant regulatory and criminal penalties** | **Special measures/penalties recommended by OV for the first offence** | **Special measures/penalties recommended by OV if the offence is repeated** | **Judicial decisions/ notes** |
| --- | --- | --- | --- | --- |
| Section 4 of the German Animal Welfare Act;  Section 12(1) of the German Ordinance on the Protection of Animals in Connection with Slaughter or Killing | According to Section 18(1)(5) of the German Animal Welfare Act, any person who deliberately or negligently kills a vertebrate in contravention of Section 4 (1 shall be guilty of a regulatory offence  According to Section 17(2)(a) and/or (b) of the German Animal Welfare Act, any person who commits an offence out of cruelty or if the animal was subjected to prolonged or repeated significant pain or suffering, shall be liable to up to three years imprisonment or a fine | Issue an animal protection order which mandates the prohibition of slaughter until standard operating procedures and practices are updated, and mandate to check the effectiveness of stunning for every animal  Appropriate range for a regulatory fine: €500.00 to €1,000.00  File a criminal complaint | If an employee has intentionally or negligently caused significant pain or suffering to an animal, an animal protection order should be issued to prohibit the employee from continuing to work in this or any other relevant position, until they have undergone re-training and have provided proof of acquiring the necessary expertise  If the person concerned no longer demonstrates sufficient competence, knowledge, or sense of responsibility to carry out relevant tasks, their certificate of competence according to Article 21 of Regulation (EC) No 1099/2009 may be temporarily or permanently withdrawn  Appropriate range for a regulatory fine:  €1,000.00 to €2,000.00  File a criminal complaint | Punished as criminal offences in a past case:  Serious deficiencies in electrical stunning of pigs persisted over a period of two years, resulting in an unacceptably high rate of incorrect stunning. This caused pigs to show signs of consciousness and pain during slaughter. Additionally, re-stunning was often carried out incorrectly.  A fine of 120 daily rates was imposed on the abattoir operator for this offence.  Source: District Court of Kassel,  Court verdict from April 27^th^, 2020,  Reference number: 9 Ns - 9634 Js 23170/13  The following violation resulted in the imposition of a regulatory fine:  An undetermined number of animals were slaughtered without adequate stunning, resulting in violent limb movements during hanging. These offenses, along with multiple other violations of food and animal protection regulations, were penalised with a significant fine.  Source: Kiel District Court,  Court order from 19.12.2018,  Reference number: 590 Js 10044/16 |

**Offense (37):** The time limit allowed between stunning and bleeding is exceeded without a certificate of exemption (in accordance with Section 13(2) of the German Ordinance on the Protection of Animals during Slaughter or Killing)

| **Citation of legal/regulatory requirements to protect animals at the time of slaughter/killing** | **Citation of relevant regulatory and criminal penalties** | **Special measures/penalties recommended by OV for the first offence** | **Special measures/penalties recommended by OV for the event of failure to remedy the defect by the deadline or in the event of repeated offence** | **Judicial decisions/ notes** |
| --- | --- | --- | --- | --- |
| Section 12(6)(1) of the German Ordinance on the Protection of Animals in Connection with Slaughter or Killing | According to Section 18(1)(3)(b) of the German Animal Welfare Act in conjunction with Section 16(2)(3) of the German Ordinance on the Protection of Animals in Connection with Slaughter or Killing, any person who deliberately or negligently does not commence bleeding in a timely manner during the period specified in column 2 of Appendix 2 in contravention of 12(6)(1) shall be guilty of a regulatory offence  According to Section 17(2)(a) and/or (b) of the German Animal Welfare Act, any person who commits an offence out of cruelty or if the animal was subjected to prolonged or repeated significant pain or suffering, shall be liable to up to three years imprisonment or a fine | Issue an animal protection order which mandates the prohibition of slaughter until compliance with the time prescribed in Annex 2, column 2 of the German Ordinance on the Protection of Animals in Connection with Slaughter or Killing is ensured; if necessary, mandate to optimise the technique use (e.g. speed of the winch, mandate to perform bleeding while lying down, improvement/easement of the slinging technique); if necessary, consult external assessors; request for an assessment of the permitted slaughter speed by the licensing authority; if necessary, reduce the slaughter speed  Appropriate range for a regulatory fine:  €150.00 to €400.00 | Appropriate range for a regulatory fine/periodic penalty payment:  €300.00 to €800.00  File a criminal complaint | The following violation resulted in the imposition of a regulatory fine:  35 animals were not bled immediately after stunning because the stunning traps were not opened. As a result, the time between stunning and the start of bleeding was between 60 and 76 seconds, which made it impossible to ensure that brain activity was extinguished. These violations, along with multiple other violations of food and animal protection regulations, resulted in a substantial fine.  Source: Kiel District Court,  Court order from December 19^th^, 2018,  Reference number: 590 Js 10044/16 |

**Offense (38):** Further preparation or scalding of slaughtered animals (such as removing the head, eyes, or ears) is performed while the animal is still showing signs of consciousness

| **Citation of legal/regulatory requirements to protect animals at the time of slaughter/killing** | **Citation of relevant regulatory and criminal penalties** | **Special measures/penalties recommended by OV for the first offence** | **Special measures/penalties recommended by OV if the offence is repeated** | **Judicial decisions/ notes** |
| --- | --- | --- | --- | --- |
| Section 12(7)(1) of the German Ordinance on the Protection of Animals in Connection with Slaughter or Killing; Article 15(1) in conjunction with Annex III (3.2)(3) of Regulation (EC) No 1099/2009 | According to Section 18(1)(3)(b) of the German Animal Welfare Act in conjunction with Section 16(2)(7) of the German Ordinance on the Protection of Animals in Connection with Slaughter or Killing, any person who deliberately or negligently carries out further preparation or scalding of slaughtered animals in contravention of Section 12(7)(1) shall be guilty of a regulatory offence  According to Section 18(3)(2)(b) of the German Animal Welfare Act in conjunction with Section 16(4)(9) of the German Ordinance on the Protection of Animals in Connection with Slaughter or Killing, any person who deliberately or negligently does not ensure that any further preparation or scalding is carried out only after it has been checked that there are no longer any signs of life in the animal in contravention of Article 15(1) in conjunction with Annex III (3.2)(3) shall be guilty of a regulatory offence | Issue an animal protection order which mandates to monitor the stunning effectiveness of every animal; if necessary, allow the person to work under the supervision of an experienced employee with a certificate of competence for a certain period of time (e.g. 3 months)  Investigate whether the operator of the abattoir violated Section 130 of the German Act on Regulatory Offences  Appropriate range for a regulatory fine:  €500.00 to €1,000.00 €  File a criminal complaint | Appropriate range for a regulatory fine/periodic penalty payment:  €1,000.00 to €2,000.00  File a criminal complaint |  |

| **Citation of legal/regulatory requirements to protect animals at the time of slaughter/killing** | **Citation of relevant regulatory and criminal penalties** | **Special measures/penalties recommended by OV for the first offence** | **Special measures/penalties recommended by OV if the offence is repeated** | **Judicial decisions/ notes** |
| --- | --- | --- | --- | --- |
|  | According to Section 17(2)(a) and/or (b) of the German Animal Welfare Act, any person who commits an offence out of cruelty or if the animal was subjected to prolonged or repeated significant pain or suffering, shall be liable to up to three years imprisonment or a fine |  |  |  |

# Part 6: Measures and Penalties for employees performing tasks without an appropriate certificate of competence

**Offense (39):** Handling and caring for animals prior to stunning is performed by an unqualified person without the required certificate of competence

| **Citation of legal/regulatory requirements to protect animals at the time of slaughter/killing** | **Citation of relevant regulatory and criminal penalties** | **Special measures/penalties recommended by OV for the first offence** | **Special measures/penalties recommended by OV if the offence is repeated** | **Judicial decisions/ notes** |
| --- | --- | --- | --- | --- |
| Article 7(2)(a) of Regulation (EC) No 1099/2009 | According to Section 18(3)(2)(a) of the German Animal Welfare Act in conjunction with Section 16(3)(1) of the German Ordinance on the Protection of Animals in Connection with Slaughter or Killing, any person who deliberately or negligently does not ensure that handling and caring for animals prior to restraint is carried out by a person who holds an appropriate certificate of competence in contravention of Article 7(2)(a), shall be guilty of a regulatory offence | Issue an animal protection order against the abattoir which mandates to only allow persons with a certificate of competence to handle and care for slaughter animals  Issue an animal protection order against the employee without the appropriate certificate of competence which, if appropriate, prohibits handling and caring for animals or only permit the employee to perform these tasks under supervision  Investigate whether the operator of the abattoir violated Section 130 of the German Act on Regulatory Offences  Regulatory fine | Periodic penalty payment/regulatory fine |  |

**Offense (40):** Stunning, killing, and related tasks are carried out by an individual without the required certification of competence

| **Citation of legal/regulatory requirements to protect animals at the time of slaughter/killing** | **Citation of relevant regulatory and criminal penalties** | **Special measures/penalties recommended by OV for the first offence** | **Special measures/penalties recommended by OV if the offence is repeated** | **Judicial decisions/ notes** |
| --- | --- | --- | --- | --- |
| Article 7(2)(b, c, d, e, f or g) of Regulation (EC) No 1099/2009 | According to Section 18(3)(2)(b) of the German Animal Welfare Act in conjunction with Section 16(4)(1) of the German Ordinance on the Protection of Animals in Connection with Slaughter or Killing, any person who deliberately or negligently does not ensure that the task is only carried out by a person who has a certificate of competence referred to therein in contravention of Article 7(2)(b, c, d, e, f or g), shall be guilty of a regulatory offence  According to Section 17(2)(a) and/or (b) of the German Animal Welfare Act, any person who commits an offence out of cruelty or if the animal was subjected to prolonged or repeated significant pain or suffering, shall be liable to up to three years imprisonment or a fine | Issue an animal protection order against the abattoir which mandates to only allow persons with a certificate of competence to perform killing and related activities  Issue an animal protection order against the employee without the appropriate certificate of competence which, if appropriate, prohibits him/her from performing killing and related activities until the certificate of competence has been obtained  Investigate whether the operator of the abattoir violated Section 130 of the German Act on Regulatory Offences  Appropriate range for a regulatory fine:  €430.00 to €1,100.00  File a criminal complaint | Appropriate range for a regulatory fine/periodic penalty payment:  €520.00 to €1,370.00  File a criminal complaint | The following violation resulted in the imposition of a regulatory fine:  The personnel operating the bolt gun were either inadequately trained or did not possess the required certificate of competence. Consequently, cattle were not rendered unconscious with a single, correctly placed bolt shot as mandated by the German Ordinance on the Protection of Animals in Connection with Slaughter or Killing. Instead, they had to be shot multiple times, as evidenced by eyelid blinking, which indicated that consciousness had not been extinguished. The repeated shots caused the animals to suffer pain until unconsciousness was finally achieved. These offenses, along with numerous other violations of food and animal welfare regulations, resulted in a substantial fine.  Source: Kiel Local Court,  Court order from September 28^th^, 2018,  Reference number: 590 Js 10044/16 |

We would like to thank all survey participants, official veterinarians and lawyers who supported us in preparing and completing this document.

We would like to extend a special thank you to Johanna Hahn, a PhD student in criminal law at the University of Leipzig, and to PD Dr Björnstjern Baade of Freie Universität Berlin.

We would also like to extend special thanks to the participants in our online colloquium for their expert revision of the first draft.

Project management: Dr. Susann Langforth, Univ.-Prof. Dr. Diana Meemken

Project employee: Mag. med. vet. Stephanie Schneidewind

Project duration: September 1^st^, 2021 – August 31^st^, 2022

Funding source: Veterinary Association for Animal Welfare (TVT e.V.)


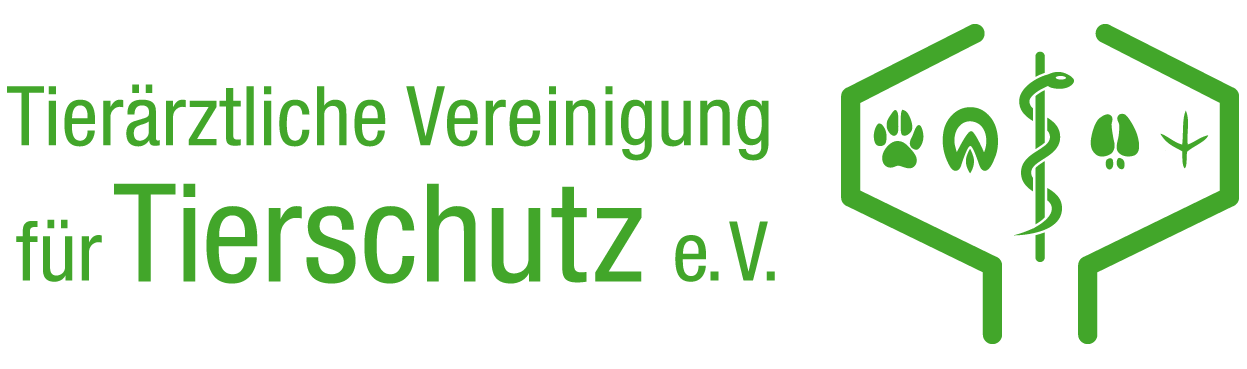

Supplement: Supplementary file 1 [file animals-13-02916-s001.zip › Supplementary Material S1_List of measures and penalties.docx]
