# Supplementary material for: Measures and Penalties for Animal Welfare Violations at German Abattoirs: A Compilation of Current Recommendations and Practices
Source: Animals (Basel). 2023 Sep 14;13(18):2916. doi: 10.3390/ani13182916 (PMC10525597; doi:10.3390/ani13182916)
Supplement: Supplementary file 1 [file animals-13-02916-s001.zip › Supplementary Material S2_ Online_Survey_English.pdf]

Online-Survey Questions (translated from German into English language)

Introduction:
Thank you for your interest in participating in this survey. The survey is specifically aimed at official veterinarians (OV) who carry out inspections at German abattoirs, as well as other individuals involved in the enforcement of animal welfare laws and regulations at German abattoirs. The collected data will be used to develop a list of measures and penalties to combat animal welfare violations at abattoirs. The goal is to improve the enforcement of animal welfare legislation. This survey is part of a research project which aims to promote a uniform and predictable approach to dealing with animal welfare violations at abattoirs, thereby strengthening the role of OV in animal welfare. After completing the research project, we will send you a compiled list of measures and penalties that you can use as a reference for cases of animal welfare violations. This can especially benefit OV who are new to the job.

Procedure: In the following survey, you will be asked to provide an assessment of appropriate measures and penalties for various violations of animal welfare regulations. Additionally, data on measures taken in past animal welfare violations will be collected. This survey is concerned with the following stages in the slaughter process: driving animals (after animals have arrived at the abattoir), lairage, stunning and bleeding, as well as further processing of the slaughtered animals after bleeding. This survey does not address violations related to transportation.

We recommend filling out this survey on a PC or tablet, because the layout of many questions may not be optimal on a mobile phone. You can interrupt the survey at any time and continue later (click on the button in the upper right corner labelled "Continue Later"). Even if you do not complete the entire survey, your answers will still be included in the evaluation.

1. Did you have a full-time or part-time occupation monitoring an abattoir in the past three years? *Multiple choices allowed*
   a) Full-time occupation as an official veterinarian (OV)
   b) Part-time occupation as an OV
   c) No occupation as an OV
   d) Other occupation in law enforcement (Please describe in the comment section)
   Comment box:

2. On average, how many hours per week did you spend working in this occupation in the last three years? *Single choice allowed*
a) less than 9 hours
b) 9 to 20 hours
c) 21 to 30 hours
d) 31 to 40 hours
e) over 40 hours
   Comment box:

3. How many different abattoirs have you worked at in the last three years?
   Dropdown box: 0, 1, 2, 3, 4, 5, 6, 7, 8, 9, 10, more than 10
   Comment box:

4. In how many different abattoirs with a slaughter volume of less than 20 LU (livestock units) per week have you worked at in the past three years? (One LU = approximately 500 kg live weight)
   Dropdown box: 0, 1, 2, 3, 4, 5, 6, 7, 8, 9, 10, more than 10
   Comment box:

5. In how many different abattoirs with a slaughter volume of 20 to 100 LU per week have you worked at in the past three years?
   Dropdown box: 0, 1, 2, 3, 4, 5, 6, 7, 8, 9, 10, more than 10
   Comment box:

6. In how many different abattoirs with a slaughter volume of more than 100 LU per week have you worked at in the past three years?
   Dropdown box: 0, 1, 2, 3, 4, 5, 6, 7, 8, 9, 10, more than 10
   Comment box:

7. Which animal species were slaughtered in the abattoirs you monitored in the past three years?
   a) Pigs

b) Cattle
c) Goat
d) Sheep
e) Horse
f) Poultry
g) Hares/rabbits
h) Other/Comments
Comment box:

8. Which stunning methods were used in the abattoirs you worked at in the past three years?
a) Captive bolt stunning
b) Electrical stunning
c) Carbon dioxide stunning
d) Water bath stunning
i) Other stunning methods/Comments
Comment box:

9. What else would you like to tell us about your occupation (specific tasks in the official veterinary department, etc.) (optional):
Comment box:

10. How many years of work experience do you have in this role?
a) 1 - 3 years
b) 4 - 7 years
c) 8 - 11 years
d) 12 - 15 years
e) 16 - 19 years
f) 20 - 23 years
g) 24 - 27 years
h) 28 - 31 years
i) 32- 35 years
j) More than 36 years

The following two questions will be asked for all animal welfare violations included in this survey (referred to as a "case" from now on):

1) What measure(s) and penalties would be appropriate in this case (for the first violation as well as for a repeated violation)?
2) Has such a case occurred in your work environment? If yes, what measures and penalties were ordered? In your opinion, were the ordered measures appropriate?

"First Violation": The abattoir/employee violates a specific law/regulation for the first time.

"Repeated Violation": The violation has been committed by the same abattoir/employee before, meaning there is already a written record of the violation.

Please feel free to let us know in the comment field whom the measures should affect (e.g., the abattoir operator, employee, animal welfare officer).

There are no mandatory questions in this survey. The comment fields can be used to provide additional information if desired. Additional comments on the case itself are also welcome but not mandatory.

There is no "right" or "wrong" in answering these questions – we want to capture your experiences and assessments to establish an expert consensus in this area.

In total, 22 cases will be presented in this questionnaire.

This survey will take approximately 45 minutes to 1 hour. In return, you will receive a list of measures and penalties this year, and you will make a significant contribution to animal welfare.

Case Number	Description of the animal welfare violation
1	As a cow resists walking into a stunning box by standing still, an abattoir employee consciously administers multiple electric shocks to the anus of the cow within 5 seconds, using an electric driving device. To avoid the pain this inflicts, the cow continues to walk into the stunning box. This action violates Section 5(1)(1) of the German Ordinance on the Protection of Animals in Connection with Slaughter or Killing in conjunction with Annex III(1.9) of Regulation (EC) No 1099/2009.
2	An abattoir employee applies electric shocks on the thigh muscles of a healthy calf in the single file chute (age: approximately 20 weeks), without attempting to drive the animal with alternative driving aids beforehand. The calf reacts to the first electric shock (which lasted less than one second) by moving forward into the killing trap. This action violates Section 5(1)(1) of the German Ordinance on the Protection of Animals in Connection with Slaughter or Killing in conjunction with Annex III(1.9) of Regulation (EC) No 1099/2009.
3	In the driving lane to the killing trap, an abattoir employee strikes a cow on the left eye with a driving stick twice, which results in a moderate swelling within a few minutes. This action violates Article 15(1) in conjunction with Annex III(1.8)(a) and (b) of Regulation (EC) No 1099/2009.
4	While attempting to escape stunning, a calf gets its head wedged between two metal posts. In order to free the calf from this position, an abattoir employee forcefully kicks the calf's head towards the ground. This action violates Article 15(1) in conjunction with Annex III(1.8)(a) and (b) of Regulation (EC) No 1099/2009.
5	As a cow resists entering a stunning box by standing still, an abattoir employee twists the tail of the animal 180 degrees. To escape the pain caused by this, the cow continues to move into the stunning box. This action violates Article 15(1) in conjunction with Annex III (1.8.)(e) of Regulation (EC) No 1099/2009.
6	A truck loaded with approximately 5000 broiler chickens departed from the origin farm at 01:00 AM and arrived at the abattoir premises at 7:00 AM. The animals remained in their containers in the transport vehicle until 11:00 AM. At 11:00 AM, the animals were immediately sent for slaughter. Upon arrival at the abattoir, the animals were not provided with water. The outside temperature was around 7°C in the morning and approximately 15°C around 11:00 AM. The stocking density of the containers met the minimum requirements of Annex 1 of the Animal Welfare Transport Regulation. This action violates Section 7(2)(2) of the German Ordinance on the Protection of Animals in Connection with Slaughter or Killing as well as Section 10(1) and (3) of the Animal Transport Protection Law (EG) No. 1/2005.

7	During the unloading of transport containers from a transport vehicle, an abattoir employee repeatedly drops containers with live rabbits (first onto the ground, then onto another container) to accelerate the unloading process. As a result of the impact, the animals show clear signs of distress. This violates Article 15(1) in conjunction with Annex III (1.3.)(1)(a) of Regulation (EC) No 1099/2009.
8	Because a pig starts moving backward while being driven into the stunning box, an abattoir employee lowers the rear gate onto the back of the animal to prevent it from moving backward further. This action causes the animal to move forward into the stunning box, and subsequently, the slaughter process continues. This behavior violates Section 3(1) of the German Ordinance on the Protection of Animals in Connection with Slaughter or Killing.
9	At the beginning of the slaughter on Monday at 5:00 am, there is no record of feeding for 28 pigs in two holding pens that were delivered the previous day at 8:00 pm. There are no traces of feed left in the trough. This violates Section 7(3) of the German Ordinance on the Protection of Animals in Connection with Slaughter or Killing.
10	In one holding pen, 10 dehorned goats are kept together with two horned goats. These animals came from different farms. This results in dominance fights, as the horned goats do not allow other animals to access the food or water trough. This violates Section 7(4) of the German Ordinance on the Protection of Animals in Connection with Slaughter or Killing.
11	At the time of inspection (11:00 am), there are 37 pigs in one holding pen and 34 animals in another holding pen. The maximum capacity for both pens is marked as 29. According to the documentation, the animals have been housed in this overcrowded condition since 8:00 am. Not all animals can lie down, stand up, and lie down freely. This violates Section 8(2)(1) of the German Ordinance on the Protection of Animals in Connection with Slaughter or Killing.
12	A sheep is not stunned before the beginning of exsanguination. There is no official exemption permit for slaughtering without stunning (ritual slaughter). The sheep is killed while fully conscious through a cut to the throat. The cut is made with a sharp knife, reaching the spinal cord. This violates Section 4a(2)(2) of the German Animal Welfare Act.
13	In an abattoir, the electrical stunning of fattening pigs is carried out using a stunning tong as follows: first, the head is electrified for approximately 10 seconds, and immediately afterward, the tongs are relocated and a heart electrification is performed for approximately 8 seconds. The employee places the electrodes of the stunning tongs at the jaw joint of all animals observed during the inspection. Some animals show signs of insufficient stunning after the heart electrification. This violates Section 12(1) of German Ordinance on the Protection of Animals in Connection with Slaughter or Killing.
14	In a cattle abattoir, out of 50 observed cattle, 5 are not bled while hanging within the legally prescribed time. Despite the competent employee's diligent efforts, (s)he is unable to complete his/her tasks (checking for stunning effectiveness, slinging, and hoisting the animals) within the legally required time in these 5 cases. The maximum duration of 60 seconds is exceeded by approximately 5 seconds in two cases and by approximately 10 seconds in the remaining 3 cases. During bleeding, the 5 affected animals show no signs of questionable stunning. There is no exemption permit according to Section 13(2) of the German Ordinance on the Protection of Animals in Connection with Slaughter or Killing for exceeding the permissible duration between stunning and bleeding. This violates Section 12(6)(1) of the German Ordinance on the Protection of Animals in Connection with Slaughter or Killing.
15	An agitated grazing cattle is planned to be unloaded in a family abattoir (less than 20 livestock units per week). The competent butcher intentionally uses a captive bolt shot to the neck of the animal to immobilize it as he believes he cannot safely stun it otherwise. The cattle collapses and is then subjected to

	slaughter. The butcher promptly administers a correct captive bolt shot to the head for stunning. This violates Article 15(3)(c) of Regulation (EC) No 1099/2009.
16	In a cattle abattoir, the stunning effectiveness of the captive bolt stunning is being monitored. There are indications of ineffective stunning in some individual animals, and in all cases, prompt and proper re-stunning is carried out. The stunning box has a head fixation, ensuring that the correct positioning is achieved for each animal. The captive bolt device is heavily notched. This violates Section(12)(5)(1) of the German Ordinance on the Protection of Animals in Connection with Slaughter or Killing in conjunction with Article 9 (1) and (2) of Regulation (EC) No 1099/2009.
17	To slaughter a calf, the stunning box is reduced in size using a metal wedge to individually restrain the animal. However, the reduction of the box is insufficient to adequately restrain the calf. The calf makes several attempts to escape and evade, making it necessary for the abattoir employee to make multiple attempts before correctly positioning the captive bolt device. This violates Section 11(1)(1) and (2) of the German Ordinance on the Protection of Animals in Connection with Slaughter or Killing.
18	A cow is stunned with a single shot using the captive bolt stunning method. However, no stunning effectiveness check is performed on this animal. When hanging the animal, signs of inadequate stunning (e.g., directed eye movements and attempts to rise) are evident. An abattoir employee does not respond to these signs and intends to hang and bleed the animal regardless. This violates Section 4(1)(1) of the German Animal Welfare Act and Article 5(1) of Regulation (EC) No 1099/2009.
19	After scalding and singeing, you observe the carcass of a pig that is already heavily reddened from the outside. The body cavity of the hanging animal is opened, and the animal bleeds heavily. Upon inspecting the hanging animal, you notice that there is no bleeding incision on its neck. For the other animals slaughtered on that day (50 animals), a bleeding incision is present. This violates Section 12(7) of the German Ordinance on the Protection of Animals in Connection with Slaughter or Killing.
20	A transporter delivers several cattle to an abattoir. One of the cattle appears to be stuck on the floor of the truck, with its hind limbs splayed out. Without attempting to lift the animal, the transporter immediately uses a chain loop and attaches it to one of the hind limbs. After hooking it up to a winch, the animal is pulled into the anteroom of the abattoir in a prone position without stunning. This violates Annex III(1.11) of Regulation (EC) No 1099/2009 and Section 8(1)(3) of the German Ordinance on the Protection of Animals in Connection with Slaughter or Killing.
21	During the unloading of a group of pigs at the abattoir, one of the pigs exhibits significant lameness and a fresh, open, deep wound over 5 cm in size on one of its hind limbs. This particular pig is grouped together in a holding pen with the unaffected pigs from the same origin farm and is not prioritized for slaughter. This violates Section 8(1)(1) and (2) of the German Ordinance on the Protection of Animals in Connection with Slaughter or Killing.
22	You (as the official veterinarian) request the submission of an official certificate of expertise from an employee who performs tasks related to herding, stunning, and bleeding. The presented proof of expertise covers herding but does not include expertise in stunning and bleeding. This violates Section (4)(1) of the German Ordinance on the Protection of Animals in Connection with Slaughter or Killing.

Multiple Choice (MC) Answer Options for every case:

a) Interruption and immediate correction of the faulty working method.
b) Oral instruction of the responsible employee (e.g., using a guideline).
c) Written instruction of the responsible employee.
d) Cautionary procedure according to Section 56 of the Act on Regulatory Offences without a cautionary fine.
e) Inform the supervisor of the responsible employee and/or the animal welfare officer about the incident.
f) Increase the number of inspections in the affected area of the abattoir.
g) Cautionary procedure according to Section 56 of the Act on Regulatory Offences with a fine ranging from 5-55 EUR.
h) Initiation of administrative proceedings according to Section 16a of the German Animal Welfare Act.
i) Initiation of a regulatory offence proceeding according to Section 18 of the German Animal Welfare Act.
j) Ordering a repetition of the proficiency examination according to Section 16a(1)(3) of the German Animal Welfare Act.
k) Withdrawal of the certificate of competence according to Section 4(6) of the German Ordinance on the Protection of Animals in Connection with Slaughter or Killing.
l) Filing a criminal complaint to the public prosecutor's office according to Section 41 of the German Administrative Offenses Act.

If Option (i) was selected, participants were asked to indicate a minimum and maximum value for the regulatory fine.

Additional MC answer options for question Number 2:

m) This case never occurred in my work environment.
n) This case has occurred, but no measures were ordered.

Final question: Please list the five most common animal welfare violations that have occurred at the abattoir in the past three. Please do not mention violations related to the transport to the abattoir (such as long waiting times for unloading animals from the transporter or transporting unfit animals).

1	
2	
3	
4	
5	

Thank you for your participation! You have helped us and all current and future official veterinarians in this field greatly.
