# Supplementary material for: Measures and Penalties for Animal Welfare Violations at German Abattoirs: A Compilation of Current Recommendations and Practices
Source: Animals (Basel). 2023 Sep 14;13(18):2916. doi: 10.3390/ani13182916 (PMC10525597; doi:10.3390/ani13182916)
Supplement: Supplementary file 1 [file animals-13-02916-s001.zip › Supplementary Material S3_Online_Survey_German.docx]

Original version of the online survey in German language - Originalversion der Online-Umfrage in deutscher Sprache

Vielen Dank für Ihr Interesse, an dieser Umfrage teilzunehmen. Diese Umfrage richtet sich insbesondere an amtliche Tierärzt:innen, die Überwachungstätigkeiten in deutschen Schlachtbetrieben ausüben, aber auch an weitere Personen, die mit dem Gesetzesvollzug zum Tierschutz an deutschen Schlachtbetrieben betraut sind. Anhand der erhobenen Daten soll ein Μaßnahmenkatalog zur Bekämpfung von Tierschutzverstößen im Schlachtbetrieb entwickelt werden. Ziel dabei ist es, die Tierschutzgesetzgebung durchzusetzen. Ein einheitliches und dadurch 'berechenbares' Vorgehen bei Tierschutzverstößen am Schlachthof soll hiermit gefördert werden, wodurch die Rolle der amtlichen Tierärzt:innen für den Tierschutz gestärkt werden soll. Sie werden nach Abschluss des Forschungsprojektes einen Katalog zur Verfügung gestellt bekommen, an dem Sie sich bei Tierschutzverstößen orientieren können und der neuen Kolleg:innen die Arbeit so auch erleichtert. Ablauf: Im folgenden Fragebogen wird um eine Einschätzung zu angemessenen Maßnahmen bei verschiedenen Verstößen gegen tierschutzrechtliche Vorschriften gebeten. Des Weiteren werden Daten zu Maßnahmen bei vergangenen Tierschutzverstößen erhoben. Diese Umfrage bezieht sich auf die folgenden Abschnitte im Schlachtprozedere: Treiben (ab Anlieferung der Tiere am Schlachtbetrieb), Unterbringung im Wartestall, Betäubung und Entblutung sowie weitere Verarbeitung der Schlachttiere nach der Entblutung (NICHT auf den Transport). Wir empfehlen, diese Umfrage am PC oder Tablet durchzuführen, da die Ansicht vieler Fragen auf dem Mobiltelefon ungünstig ist. Sie können die Umfrage jederzeit unterbrechen und später fortführen (klicken Sie dazu den Knopf oben rechts namens "Später Fortfahren"). Uns ist auch damit geholfen, wenn Sie diese Umfrage nicht vollständig durchführen. Ihre Antworten werden donnoch in die Bewertung fließen.

1. Haben Sie in den vergangenen drei Jahren eine hauptamtliche oder nebenamtliche Tätigkeit im Bereich der Schlachttier- und Fleischuntersuchung ausgeführt? *Mehrfachnennungen möglich.*
   a) hauptamtliche Tätigkeit
2. nebenamtliche Tätigkeit
3. keine amtliche Tätigkeit
4. keine Tätigkeit in der Schlachttier- und Fleischuntersuchung (bitte Tätigkeit im Kommentarfeld beschreiben)

Kommentarfeld:

|  |
| --- |

1. Wie viele Stunden pro Woche betrug durchschnittlich Ihr Arbeitsumfang in dieser Tätigkeit in den letzten drei Jahren? *Einfachauswahl.*
2. weniger als 9 Stunden
3. 9 bis 20 Stunden
4. 21 bis 30 Stunden
5. 31 bis 40 Stunden
6. über 40 Stunden

Kommentarfeld:

|  |
| --- |

1. An wie vielen unterschiedlichen Schlachtbetrieben haben Sie in den letzten drei Jahren gearbeitet?
   *Dropdown: 0, 1, 2, 3, 4, 5, 6, 7, 8, 9, 10, über 10*

Kommentarfeld:

|  |
| --- |

1. In wie vielen unterschiedlichen Schlachtbetrieben mit einem Schlachtvolumen mit weniger als 20 GVE pro Woche haben Sie in den vergangenen drei Jahren gearbeitet? (Eine GVE = circa 500 kg Lebendgewicht)
   *Dropdown: 0, 1, 2, 3, 4, 5, 6, 7, 8, 9, 10, über 10*
   Kommentarfeld:

|  |
| --- |

1. In wie vielen unterschiedlichen Schlachtbetrieben mit einem Schlachtvolumen von 20 bis 100 GVE/Woche haben Sie in den vergangenen drei Jahren gearbeitet? (Eine GVE = circa 500 kg Lebendgewicht)

*Dropdown: 0, 1, 2, 3, 4, 5, 6, 7, 8, 9, 10, über 10*
Kommentarfeld:

|  |
| --- |

1. In wie vielen unterschiedlichen Schlachtbetrieben mit einem Schlachtvolumen von mehr als 100 GVE/Woche haben Sie in den vergangenen drei Jahren gearbeitet? (Eine GVE = circa 500 kg Lebendgewicht)
   *Dropdown: 0, 1, 2, 3, 4, 5, 6, 7, 8, 9, 10, über 10*
   Kommentarfeld:

|  |
| --- |

1. Welche Tierarten wurden in den Betrieben geschlachtet, in denen Sie in den vergangenen drei Jahren tätig waren? *Mehrfachnennungen möglich.*
   a) Schwein
2. Rind
3. Ziege
4. Schaf
5. Pferd
6. Geflügel
7. Hasenartige
8. Sonstiges/Kommentar
9. Kommentarfeld:

|  |
| --- |

1. Welche Betäubungsmethoden wurden in den Betrieben verwendet, in denen Sie in den vergangenen drei Jahren tätig waren? *Mehrfachnennungen möglich.*
2. Bolzenschussbetäubung
3. Elektrobetäubung
4. Kohlendioxidbetäubung
5. Wasserbadbetäubung
6. Sonstige Betäubungsmethoden/Kommentar

Kommentarfeld:

|  |
| --- |

1. Was möchten Sie uns noch über Ihre Tätigkeit mitteilen (genauer Aufgabenbereich im Veterinäramt, etc.) (optional):
   Kommentarfeld:

|  |
| --- |

1. Wie viele Jahre Arbeitserfahrung haben Sie in dieser Tätigkeit?
   a) 1 - 3 Jahre
2. 4 - 7 Jahre
3. 8 - 11 Jahre
4. 12 - 15 Jahre
5. 16 - 19 Jahre
6. 20 - 23 Jahre
7. 24 - 27 Jahre
8. 28 - 31 Jahre
9. 32- 35 Jahre
10. More than 36 Jahre

Im Folgenden werden zu einem Tierschutzverstoß (ab jetzt nur noch „Fall“ genannt) zwei Fragen gestellt:

1. Welche Maßnahme(n) wären aus Ihrer Sicht bei diesem Fall (beim ersten Verstoß sowie beim wiederholten Verstoß) angemessen?

2. Ist solch ein Fall in Ihrem Arbeitsumfeld aufgetreten? Falls ja, welche Maßnahmen wurden angeordnet? Waren dieangeordneten Maßnahmen Ihrer Ansicht nach angemessen?

„Erster Verstoß“: Der Betrieb/(der/die) Mitarbeiter:in wird das erste Mal mit diesem Verstoß auffällig

„Wiederholter Verstoß“: Der Verstoß wurde im Vorfeld schon mal festgestellt, d.h. gibt es schon eine schriftliche Mängelfeststellung.

Teilen Sie uns im Kommentarfeld gern mit, gegen wen sich die Maßnahmen richten sollten (Schlachthofbetreiber:in, -mitarbeiter:in, Tierschutzbeauftragte:r)

Es gibt in dieser Umfrage keine Pflichtfragen. Die Kommentarfelder können dazu verwendet werden, weitere Angaben zu machen, wenn dies gewünscht wird. Ergänzungen/Kommentare zum Fall an sich sind ebenfalls erwünscht, aber nicht zwingend notwendig.

Es gibt kein „richtig“ oder „falsch“ bei der Beantwortung der Fragen – wir wollen Ihre Erfahrungen und Einschätzungen erfassen, um in diesem Bereich einen Expert:innenkonsens zu finden.

Insgesamt werden in diesem Fragebogen 22 Fälle abgefragt.

Diese Umfrage dauert circa 45 min - 1 h. Dafür erhalten Sie jedoch noch in diesem Jahr einen Maßnahmenkatalog und leisten einen bedeutenden Beitrag zum Tierschutz.

| Fallnr. | Fallbeschreibung |
| --- | --- |
| 1 | Da sich ein Rind gegen den Zutritt in eine Betäubungsbox durch Stehenbleiben widersetzt, verabreicht ein:e Schlachthofmitarbeiter:in innerhalb von 5 Sekunden mehrmals bewusst Stromstöße mittels eines elektrischen Treibgerätes im Analbereich des Rindes. Um dem dadurch entstandenen Schmerz zu entkommen, läuft das Rind weiter in die Betäubungsbox. Dies verstößt gegen § 5 Absatz 1 TierSchlV i.V.m. VO (EG) Nr. 1099/2009. |
| 2 | Bei der Vereinzelung zur Tötungsfalle wird ein elektrisches Treibgerät einmal auf der Zutriebsstrecke bei einem gesunden Kalb (Alter: circa 20 Wochen) an der Oberschenkelmuskulatur verwendet, ohne davor einen Treibversuch mit einer anderen Treibhilfe zu unternehmen. Das Kalb reagiert auf den ersten Stromstoß (wobei die Dauer des Stromstoßes weniger als eine Sekunde beträgt) mit Vorwärtsgehen in die Tötungsfalle.  Dies verstößt gegen § 5 Absatz 1 TierSchlV i.V.m. VO (EG) Nr. 1099/2009. |
| 3 | Im Treibgang zur Tötungsfalle schlägt ein:e Schlachthofmitarbeiter:in einem nachdrängenden Rind zwei Mal mit einem Treibstock gegen das linke Auge, welches innerhalb von wenigen Minuten eine mittelgradige Schwellung aufweist. Dies verstößt gegen Artikel 15 Absatz 1 i.V.m. Anhang III Nummer 1.8. Buchstabe a und b der VO (EG) Nr. 1099/2009. |
| 4 | Bei dem Versuch, der Betäubung zu entkommen, gerät ein Kalb mit seinem Kopf zwischen zwei Metallpfosten, wo es sich verkeilt. Um das Kalb aus dieser Lage herauszubekommen, tritt ein:e Schlachthofmitarbeiter:in den Kopf des Kalbes gewaltsam in Richtung Boden. Dies verstößt gegen Artikel 15 Absatz 1 i.V.m. Anhang III Nummer 1.8. Buchstabe a und b der VO (EG) Nr. 1099/2009. |
| 5 | Da sich ein Rind gegen den Zutritt in eine Betäubungsbox durch Stehenbleiben widersetzt, verdreht ein:e Schlachthofmitarbeiter:in den Schwanz des Tieres um 180 Grad. Um dem dadurch entstandenen Schmerz zu entkommen, geht das Rind weiter in die Betäubungsbox. Dies verstößt gegen Artikel 15 Absatz 1 i.V.m. Anhang III Nummer 1.8. Buchstabe e der VO (EG) Nr. 1099/2009. |
| 6 | Ein LKW mit circa 5000 geladenen Masthähnchen fuhr um 01:00 Uhr vom Herkunftsbetrieb ab und kam um 7:00 Uhr auf dem Gelände eines Schlachtbetriebes an. Bis 11:00 Uhr verblieben die Tiere in ihren Behältnissen im Transportfahrzeug. 11:00 Uhr wurden die Tiere unmittelbar der Schlachtung zugeführt. Nach Ankunft am Schlachtbetrieb wurden die Tiere nicht mit Wasser versorgt. Die Außentemperatur betrug morgens circa 7°C und gegen 11:00 etwa 15°C. Die Belegdichte der Behältnisse entspricht den Mindestanforderungen der Anlage 1 der TierSchTrV. Dies verstößt gegen § 7 Abs. 2 Satz 2 TierSchlV sowie gegen § 10 Absatz 1 und 3 TierSchTrV. |
| 7 | Beim Entladen der Transportbehältnisse aus einem Transportfahrzeug lässt ein:e Schlachthofmitarbeiter:in mehrmals hintereinander die Behältnisse mit lebendigen Schlachtkaninchen fallen (erst auf den Boden, dann auf ein weiteres Behältnis), um den Entladevorgang zu beschleunigen. Dabei ist nach Aufprall der Behältnisse eine deutliche Erregung der Tiere zu erkennen. Dies verstößt gegen Artikel 15 Absatz 1 i.V.m. Anhang III Nummer 1.3. Buchstabe a. |
| 8 | Weil ein Schwein beim Eintrieb in die Betäubungsbox beginnt rückwärts zu gehen, senkt ein:e Schlachthofmitarbeiter:in das hintere Tor auf den Rücken des Tieres ab, um das weitere Rückwärtsgehen des Tieres zu verhindern. Dies veranlasst eine Vorwärtsbewegung des Tieres in die Betäubungsbox und anschließend wird der Schlachtprozess fortgesetzt. Dies verstößt gegen § 3 Absatz 1 TierSchlV. |
| 9 | Zu Beginn der Schlachtung am Montag um 5:00 Uhr morgens ist zu 28 Schweinen in zwei Wartebuchten, die schon am Vortag um 20:00 Uhr angeliefert worden waren, keine Fütterung vermerkt. Es sind keinerlei Futterreste im Trog zu sehen. Dies verstößt gegen § 7 Absatz 3 TierSchlV. |
| 10 | In einer Wartebucht werden 10 unbehornte Ziegen gemeinsam mit zwei behornten Ziegen untergebracht. Diese Tiere stammen aus unterschiedlichen Herkunftsbetrieben. Es kommt zu Rangordnungskämpfen. Die behornten Ziegen lassen keine direkten Nachbarn am Futter- oder Wassertrog zu. Dies verstößt gegen § 7 Absatz 4 TierSchlV. |
| 11 | In einem Wartestall sind in einer Bucht zum Zeitpunkt der Kontrolle (11:00 Uhr) 37 Schweine eingestallt, in einer zweiten Bucht 34 Tiere. Die Maximalbelegung beider Buchten ist mit 29 gekennzeichnet. Laut Dokumentation sind die Tiere seit 8:00 Uhr in dieser Belegdichte untergebracht. Nicht alle Tiere können ungehindert liegen, aufstehen und sich hinlegen. Dies verstößt gegen § 8 Absatz 2 Punkt 1 TierSchlV. 1. |
| 12 | Ein Schaf wird vor Beginn des Blutentzugs nicht betäubt, ohne dass eine behördliche Ausnahmengenehmigung zum betäubungslosen Schlachten (Schächten) vorliegt. Das Schaf wird bei vollem Bewusstsein durch einen Kehlschnitt getötet. Dabei wird der Kehlschnitt mit einem scharfen Messer bis zur Wirbelsäule durchgeführt. Dies verstößt gegen § 4a Absatz 2 Nummer 2 TierSchG. |
| 13 | In einem Schlachtbetrieb wird die Elektrobetäubung von Mastschweinen anhand einer Betäubungszange folgendermaßen durchgeführt: erst erfolgt die Kopfdurchströmung für circa 10 Sekunden und direkt im Anschluss wird die Zange umgesetzt und eine Herzdurchströmung für circa 8 Sekunden durchgeführt. Der Mitarbeiter setzt bei allen während der Kontrolle beobachteten Tiere die Elektroden der Betäubungszange am Kiefergelenk an. Dabei zeigen Tiere vereinzelt nach der Herzdurchströmung Anzeichen einer mangelhaften Betäubung. Dies verstößt gegen § 12 Absatz 1 TierSchlV. |
| 14 | In einem Rinderschlachtbetrieb werden 5 von 50 beobachteten Rindern nicht innerhalb der gesetzlich vorgeschriebenen Zeit im Hängen entblutet. Der sachkundige Mitarbeiter schafft es in diesen 5 Fällen trotz seiner zügigen Arbeitsweise nicht, seine Aufgaben (Betäubungserfolgskontrolle, Anschlingen und Hochziehen der Tiere) innerhalb der rechtlich vorgegebenen Zeit zu erfüllen. Die Höchstdauer von 60 Sekunden wird in zwei Fällen mit circa 5 Sekunden überschritten, und in den übrigen 3 Fällen mit circa 10 Sekunden. Beim Entbluten zeigen die 5 betroffenen Tiere keine Anzeichen einer fraglichen Betäubung. Für eine Überschreitung der zulässigen Dauer zwischen Betäubung und Entblutung liegt keine Ausnahmegenehmigung nach § 13 Absatz 2 TierSchlV vor. Dies verstößt gegen § 12 Absatz 6 Satz 1 TierSchlV. 1. |
| 15 | Ein unruhiges Weiderind soll in einem Familienschlachtbetrieb (unter 20 GVE pro Woche) entladen werden. Der sachkundige Fleischer setzt bewusst einen Bolzenschuss in den Nacken des Rindes, um es zu immobilisieren, da er der Meinung ist, es sonst nicht sicher betäuben zu können. Das Rind bricht zusammen und wird der Schlachtung zugeführt. Er betäubt unverzüglich mit einem korrekten Bolzenschuss in den Schädel nach. Dies verstößt gegen Artikel 15 Absatz 3 Buchstabe c VO (EG) Nr. 1099/2009. |
| 16 | In einem Rinderschlachtbetrieb wird die Betäubungseffektivität der Bolzenschussbetäubung überprüft. Bei einzelnen Tieren gibt es Hinweise auf eine Fehlbetäubung, wobei in beiden Fällen unverzüglich fachgerecht nachbetäubt wird. Die Betäubungsfalle verfügt über eine Kopffixierung, wodurch der Ansatz bei jedem Tier korrekt durchgeführt werden kann. Bei der Überprüfung des Bolzenschussgerätes fällt auf, dass der Bolzen stark gekerbt ist. Dies verstößt gegen § 12 Absatz 5 Nummer 1 TierSchlV i.V.m. Artikel 9 Absatz 1 und 2 der Verordnung (EG) Nr. 1099/2009. |
| 17 | Für die Schlachtung eines Kalbs wird die Betäubungsbucht mit einem Metallkeil verkleinert, um das Tier einzeln zu fixieren. Die Verkleinerung der Bucht reicht allerdings nicht aus, um das Kalb ausreichend zu fixieren. Das Kalb unternimmt mehrmals Flucht- und Ausweichversuche, wodurch es dem Schlachthofmitarbeiter erst nach mehreren Versuchen gelingt, den Bolzenschussapparat korrekt anzusetzen. Dies verstößt gegen § 11 Absatz 1 Satz 1 und 2 TierSchlV. |
| 18 | Ein Rind wird per Bolzenschussbetäubung mit einem Schuss betäubt. Bei diesem Tier wird keine Betäubungserfolgskontrolle durchgeführt. Beim Aufhängen des Tieres sind Anzeichen einer mangelhaften Betäubung (z.B. gerichtete Augenbewegungen und Aufrichtversuche) zu erkennen. Ein:e Schlachthofmitarbeiter:in reagiert nicht auf die Anzeichen und möchte das Tier aufhängen und anschließend entbluten. Dies verstößt gegen § 4 TierSchG Absatz 1 Satz 1 und Artikel 5 Absatz 1 der Verordnung (EG) Nr. 1099/2009. |
| 19 | Nach Brühen und Abflammen sehen Sie den Tierkörper eines Schweines, der bereits von außen stark rot gefärbt ist. Die Körperhöhle des hängenden Tieres wird eröffnet. Dabei blutet das Tier sehr stark. Sie begutachten das hängende Tier und bemerken, dass am Hals kein Entblutestich zu sehen ist. Bei den anderen Tieren, die an dem Tag geschlachtet wurden (50 Tiere), ist ein Entblutestich vorhanden. Dies verstößt gegen § 12 Absatz 7 TierSchlV. |
| 20 | Ein Transporteur liefert mehrere Rinder in einem Schlachtbetrieb an. Ein Rind liegt scheinbar auf dem LKW-Boden fest. Die hinteren Gliedmaßen sind ausgegrätscht. Ohne jeglichen Auftriebversuch nimmt der Transporteur sofort eine Kettenschlaufe zur Hand und befestigt diese an einer Hintergliedmaße. Nach Einhaken einer Seilwinde wird das Rind ohne Betäubung in Bauchlage in den Vorraum des Schlachthofes gezogen. Dies verstößt gegen Anhang III Nummer 1.11 der VO (EG) Nr. 1099/2009 und § 8 Absatz 1 Nummer 3 TierSchlV. |
| 21 | Bei der Entladung einer Schweinegruppe am Schlachtbetrieb fällt bei einem Schwein eine deutliche Lahmheit sowie eine frische, über 5 cm große offene, tiefe Wunde an einer Hintergliedmaße auf. Dieses Schwein wird gemeinsam mit den unauffälligen Schweinen der angelieferten Gruppe (welche vom gleichen Herkunftsbetrieb stammen) in einer Wartebucht untergebracht und nicht priorisiert getötet. Dies verstößt gegen § 8 Absatz 1 Nummer 1 und 2 TierSchlV. |
| 22 | Sie bitten (als amtlicher Tierarzt/als amtliche Tierärztin) um Vorlage eines amtlichen Sachkundenachweises eines Mitarbeiters, welcher Tätigkeiten im Bereich des Treibens, Betäubens und Entblutens durchführt. Der vorgelegte Sachkundenachweis umfasst dabei die Sachkunde im Bereich des Treibens, jedoch nicht im Bereich des Betäubens und des Entblutens. Dies verstößt gegen § 4 Absatz 1 TierSchlV. |

Multiple Choice Answer Options für jeden Fall:

1. Unterbrechung und sofortige Korrektur der fehlerhaften Arbeitsmethode.
2. Mündliche Belehrung des verantwortlichen Mitarbeiters (z.B. mit einem Leitfaden).
3. Schriftliche Belehrung des verantwortlichen Mitarbeiters.
4. Mündliche Verwarnung des verantwortlichen Mitarbeiters nach § 56 OWiG.
5. Den Vorgesetzten des verantwortlichen Mitarbeiters und/oder den Tierschutzbeauftragten über den Vorfall informieren.
6. Häufigere Kontrollen in dem betroffenen Bereich des Schlachtbetriebs.
7. Verwarnungsverfahren nach § 56 OWiG mit einem Verwarngeld in Höhe von 5-55 EUR.
8. Einleitung eines Verwaltungsverfahrens nach § 16a TierSchG Satz 1.
9. Einleitung eines Bußgeldverfahrens wegen einer Ordnungswidrigkeit nach § 18 TierSchG in Verbindung mit § 16 TierSchlVO.
10. Anordnung einer Wiederholung der Sachkundeprüfung nach § 16a TierSchG Absatz 1 Punkt 3.
11. Entzug der Sachkunde gemäß § 4 Absatz 6 TierSchlV.
12. Abgabe als Strafanzeige an die Staatsanwaltschaft nach § 41 OWiG.

Falls Option (i) ausgewählt wurde, wurden Teilnehmende gebeten, ein angemessenes Minimum und Maximum für das Bußgeld zu nennen.

Zusätzliche Antwortoptionen bei Frage 2:

1. Dieser Fall ist nie in meinem Arbeitsumfeld vorgekommen.
2. Dieser Fall ist vorgekommen, aber es wurden keine Maßnahmen angeordnet.

Letzte Frage: Bitte nennen Sie die fünf häufigsten Tierschutzverstöße, die in den vergangenen drei Jahren am Schlachtbetrieb vorgekommen sind (im Bereich „Treiben“ (ab Anlieferung der Tiere) bis „weitere Verarbeitung der Schlachttiere“). Bitte benennen Sie keine Verstöße gegen die Tierschutztransportverordnung (wie z.B. eine lange Dauer bis zum Abladen der Tiere vom Transporter oder den Transport transportunfähiger Tiere).

| 1 |  |
| --- | --- |
| 2 |  |
| 3 |  |
| 4 |  |
| 5 |  |

Vielen Dank für Ihre Teilnahme! Sie haben uns und allen jetzigen sowie zukünftigen amtlichen Tierärzt:innen in dem Gebiet sehr geholfen.
