# Supplementary material for: Measures and Penalties for Animal Welfare Violations at German Abattoirs: A Compilation of Current Recommendations and Practices
Source: Animals (Basel). 2023 Sep 14;13(18):2916. doi: 10.3390/ani13182916 (PMC10525597; doi:10.3390/ani13182916)
Supplement: Supplementary file 1 [file animals-13-02916-s001.zip › Supplementary Material S4_ Semi structure interview with OV.docx]

Questions asked during the semi-structured interviews with official veterinarians (OV) in English and German language

English

1. Based on your personal experience in working at an abattoir, which animal welfare violations were most commonly observed, excluding those that occur during transport to the abattoir?
   1. Which measures were taken in these cases?
   2. Which measures should, from your perspective, be ordered more frequently by OV in response to the most common animal welfare violations?
2. What are the most common reasons for fines and criminal complaints concerning slaughter animals at the abattoir (again, not including violations which occur during transport to the abattoir)?
3. What is the regular procedure following a regulatory offense at the abattoir (an offense according to Section 16 of the German Ordinance on the Protection of Animals in Connection with Slaughter or Killing) - for example, in the case of improper use of an electric prod? (Please briefly explain the steps)
4. What is the regular procedure following a criminal offense (a crime according to Section 17 of the German Animal Welfare Act) at the abattoir? (Please briefly explain the steps)
5. How are the fines determined?
6. How would an OV proceed if an abattoir employee is working without the required certification of expertise?
7. What expectations/demands/wishes do you, as an OV, have for a list of measures and penalties concerning animal welfare violations at the abattoir?

German

1. Welche Tierschutzverstöße wurden in Ihrem Arbeitsumfeld am häufigsten in Schlachtbetrieben (ab der Entladung bis zur weiteren Verarbeitung der Schlachttiere) festgestellt?
   1. Welche Maßnahmen wurden in diesen Fällen angeordnet?
   2. Welche Maßnahmen sollten aus Ihrer Perspektive öfters bei den häufigsten Tierschutzverstößen von amtlichen Tierärzt:innen angeordnet werden?
2. Was sind die häufigsten Ursachen für Bußgeldanordnungen und Strafanzeigen bei Schlachttieren am Schlachthof?
3. Wie ist das reguläre Vorgehen nach einer Feststellung einer Ordnungswidrigkeit am Schlachthof (ordnungswidrig nach § 16 Tierschutz-Schlachtverordnung) – beispielsweise bei einer unzulässigen Anwendung eines Elektrotreibers? (bitte die Abläufe kurz erläutern)
4. Wie ist das reguläre Vorgehen nach einer Feststellung einer Straftat (also bei einem Verstoß gegen § 17 Tierschutzgesetz) am Schlachthof? (bitte die Abläufe kurz erläutern)
5. Wie werden die Höhen von Strafen festgesetzt?
6. Wie würde eine amtliche Tierärzt:in vorgehen, wenn ein Schlachthofmitarbeiter ohne einen entsprechenden Sachkundenachweis arbeitet?
7. Welche Erwartungen/Ansprüche/Wünsche haben Sie als amtliche Tierärzt:in an einen Maßnahmen- und Strafmaßkatalog für Tierschutzverstöße am Schlachtbetrieb?
